# Cost‐effectiveness of hepatitis C virus test‐and‐treat and risk reduction strategies among men who have sex with men living with HIV in France

**DOI:** 10.1002/jia2.26035

**Published:** 2022-11-30

**Authors:** Mathieu Castry, Anthony Cousien, Karen Champenois, Virginie Supervie, Annie Velter, Jade Ghosn, Yazdan Yazdanpanah, A. David Paltiel, Sylvie Deuffic‐Burban

**Affiliations:** ^1^ Université de Paris IAME INSERM Paris France; ^2^ Sorbonne Université Inserm Institut Pierre Louis d’Épidémiologie et de Santé Publique Paris France; ^3^ Santé publique France Saint‐Maurice France; ^4^ Service de maladies Infectieuses et tropicales Hôpital Bichat Claude Bernard Paris France; ^5^ Yale School of Public Health New Haven Connecticut USA

**Keywords:** hepatitis, cost‐effectiveness, HIV care continuum, modelling, men who have sex with men, coinfection

## Abstract

**Introduction:**

Studies suggest that hepatitis C virus (HCV) micro‐elimination is feasible among men who have sex with men (MSM) living with human immunodeficiency virus (HIV), through treatment‐as‐prevention and interventions aimed at reducing risk behaviours. However, their economic impact is poorly understood. The aim of this study was to assess the cost‐effectiveness of HCV screening and risk reduction strategies in France.

**Methods:**

A compartmental deterministic mathematical model was developed to describe HCV disease transmission and progression among MSM living with HIV in France. We evaluated different combinations of HCV screening frequency (every 12, 6 or 3 months) and risk reduction strategies (targeting only high‐risk or all MSM) from 2021 onwards. The model simulated the number of HCV infections, life‐expectancy (LYs), quality‐adjusted life‐expectancy (QALYs), lifetime costs and incremental cost‐effectiveness ratio (ICER) over a lifetime horizon (leading to an end of the simulation in 2065).

**Results:**

All strategies increased QALYs, compared with current practices, that is yearly HCV screening, with no risk reduction. A behavioural intervention resulting in a 20% risk reduction in the high‐risk group, together with yearly screening, was the least expensive strategy, and, therefore, cost‐saving compared to current practices. The ICER per QALY gained for the strategy combining risk reduction for the high‐risk group with 6‐month HCV screening, compared to risk reduction with yearly screening, was €61,389. It also prevented 398 new HCV infections between 2021 and 2065, with a cost per infection averted of €37,790. All other strategies were dominated (more expensive and less effective than some other available alternative) or not cost‐effective (ICER per QALY gained > €100,000).

**Conclusions:**

In the French context, current HCV screening practices without risk reduction among MSM living with HIV cannot be justified on economic grounds. Risk reduction interventions targeted to high‐risk individuals—alongside screening either once or twice a year—could be cost‐effective depending on the policymaker's willingness‐to‐pay.

## 1 INTRODUCTION

Since 2000, hepatitis C virus (HCV) has been an emerging epidemic among men who have sex with men (MSM) living with human immunodeficiency virus (HIV), especially in high‐income countries [1, 2]. Transmission of HCV in this population is associated with high‐risk practices, such as unprotected traumatic sex, group sex and the use of drugs [3, 4], especially the use of psychoactive substances in a sexual context (*chemsex*) [5].

The promise of highly effective and well‐tolerated direct‐acting antivirals (DAAs) [6] as a prevention tool has led the World Health Organization to issue the ambitious goal of HCV elimination by 2030 [7]. Micro‐elimination—the reduction of HCV incidence to zero in targeted (high‐risk) populations—could be a simple, low‐cost, first step towards global elimination [8]. MSM living with HIV appear to be a suitable group for micro‐elimination strategies because more than 80% of them are engaged in HIV care, where HCV monitoring is integrated. Moreover, growing awareness of HCV has recently led to more favourable conditions in France among this population, with a low prevalence of active HCV infection (1.8% in 2016 [9]) and declining primary incidence (from 0.98/100 person‐years in 2014 to 0.45/100 person‐years in 2017 [10]).

Access to care and rapid DAA treatment initiation (regardless of fibrosis stage) are universally available in France [11]. Intensified HCV screening, combined with immediate treatment, may prevent onward transmission (societal benefit) as well as HCV‐related complications (individual benefit) [12]. Indeed, early detection facilitates rapid treatment initiation leading to reduced morbidity, mortality [13] and progression to costly complications [14]. However, uncertainties persist: the cost‐effectiveness of more frequent HCV monitoring—particularly using costly polymerase chain reaction (PCR) tests (€54)—remains unproven; and DAA therapies are expensive.

Another key challenge is the reduction of risk behaviours for HCV infection [15]. Indeed, primary infection and reinfection rates among MSM who remain engaged in risky practices may particularly hamper the micro‐elimination goal. Numerous studies evaluated risk reduction interventions for HIV and other sexually transmitted infections among MSM [16, 17]. There are, however, major uncertainties regarding the potential effectiveness and related costs of HCV risk reduction in this population. Model‐based analyses can inform better decisions by providing tools to assess the costs and benefits of such strategies.

The aim of the present study was to assist decision‐makers in allocating scarce resource by evaluating the cost‐effectiveness of different HCV screening strategies (followed by immediate treatment), and risk reduction strategies among MSM living with HIV in France. Outcomes of interest included life‐expectancy (measured in life‐years, LYs), quality‐adjusted life‐expectancy (measured in quality‐adjusted life‐years, QALYs) and healthcare costs.

## 2 METHODS

### 2.1 Study design overview

We used a dynamic compartmental model. Lifetime horizon was considered, leading to an end of the simulation in 2065. All costs (in 2020 Euros €), LYs and QALYs were discounted at 2.5% per year over the first 30 years, decreasing to 1.5% thereafter [18]. We adopted a healthcare sector perspective, excluding indirect costs as recommended by the French National Authority for Health [18]. Our study population was the cohort of 67,950 French MSM living with HIV, from 2021. New individuals entered the model to take part in HCV transmission dynamics but were excluded from the economic evaluation.

### 2.2 Model description

We extended a previously published model of HCV transmission and liver disease progression among MSM living with HIV in France [12]. All individuals entered the model at the moment of HIV infection. Briefly, the states included HCV infection, HIV and HCV cascades of care (diagnosis, linkage to care and treatment), HCV disease progression and mortality (background, HIV related and HCV related). The target population was stratified into two risk groups (low‐ and high‐risk) depending on whether or not individuals engaged in chemsex. The force of HCV infection was given by the product of: (1) group‐specific transmission rates and (2) the time‐dependent HCV prevalence. We assumed homogeneous mixing, that is no mixing preferences. We assigned a relative risk of HCV transmission for the high‐risk group compared to the low‐risk group. Further model description and flow diagrams are available in Supplementary Material S1 and Figure S1. Model equations are provided in Supplementary Material S2.

### 2.3 Input parameters and model calibration

We obtained demographic and behavioural data from three main sources: (1) modelling work describing the HIV epidemic and cascade of care in France in 2010 [19], updated for 2016; (2) the French Hospital Database on HIV (ANRS CO4‐FHDH) [20] between 2014 and 2017; and (3) cross‐sectional survey PREVAGAY 2015 (conducted among French MSM attending gay venues, undertaken in 2015) [21]. We used previous estimates [19, 22] to parameterize both the initial population size in 2014 (53,200) and its distribution across the HIV cascade of care. Estimates on HIV incidence among MSM in France between 2014 and 2018 ([23]; update from [22]) allowed us to define our study population of 67,950 MSM living with HIV in 2021. The global prevalence of active HCV infection in 2014 was set at 3.62% (see Supplementary Material S1 for additional details). The proportion of MSM living with HIV engaged in chemsex practices (i.e. high‐risk group) was estimated at 28% from PREVAGAY 2015. Other parameters were derived from a search of the literature from 2017 to March 2022, with preference given to French and more recent sources. Key parameters are given in Table 1; the complete list is given in Table S1. The HCV transmission rate was calibrated via Approximated Bayesian Computation [24] to reflect decreasing HCV incidence rates observed over 2014–2017 in the ANRS CO4‐FHDH [10] (Supplementary Material S3). Treatment eligibility criteria, treatment rates and sustained virological response (SVR) rates were varied between 2014 and 2019, reflecting changes in treatment practices and DAAs availability for MSM in France (Table S2). Notably, national health insurance system data confirmed rapid treatment initiation after diagnosis (1 month) from 2019 [11].

**Table 1:** Key model parameter values

Parameter	Value	Reference	Details
HCV prevalence (RNA positive) among MSM living with HIV in 2014	3.62%	FHDH, [25]	
Primary HCV incidence among MSM living with HIV in care	2014: 0.98/100 py 2015: 0.82/100 py 2016: 0.67/100 py 2017: 0.45/100 py	[10]	FHDH hospital‐based cohort
Yearly number of new HIV infections among MSM (model entry)	2014: 2726 2015: 2634 2016: 2577 2017: 2587 2018: 2501	[23]	Estimated from surveillance data on newly diagnosed HIV cases and back‐calculation modelling (updated values using the same method as [19, 22]). Assumption after 2018: linear extrapolation of the 2014–2018 decreasing trend
Proportion of HCV coinfection among MSM with new HIV infection	2%	–	Based on surveillance data from Santé publique France [26]
Time from HIV infection to HIV diagnosis	2.7 years	[19, 22]	Estimated from surveillance data on newly diagnosed HIV cases and back‐calculation modelling. Assumption: same time interval was used for HIV‐HCV coinfection as for HIV infection
Time from HIV diagnosis to entry into HIV care	10 days	[27]	Estimated from the FHDH hospital‐based cohort for 2014–2016 (updated values using the same method as [19]). Assumption: same time interval was used for HIV‐HCV coinfection as for HIV infection
Time from entry into HCV care to HCV treatment	1 month	–	Value from 2019, based on [11]; see Table S2 for values from 2014
Efficacy of HCV treatment (DAAs)	95%		Rounded from [6]
Proportion of MSM in the high‐risk group (chemsex practices)	28%	PREVAGAY 2015	
Relative risk of HCV transmission for the high‐risk group (chemsex) compared to the low‐risk group	5.60	PREVAGAY 2015	

### 2.4 Strategies

We defined 10 strategies from 2021, based on the frequency of HCV screening (every 12, 6 or 3 months) and the implementation of a risk reduction intervention leading to a 20% reduction in the risk of HCV acquisition (Table 2). Reference scenario S1 represents current practices of yearly HCV screening, without risk reduction. S2 is the strategy currently recommended in France (6‐month HCV screening) [28]. Strategies S3 and S4 represent more intensive screening (every 3 months) for the high‐risk group or all MSM living with HIV. Strategies S5–S10 depict different combinations of screening and risk reduction. Two alternative risk reduction scenarios were presented, depending on the target population: high‐risk group (S5, S7 and S9); or all MSM living with HIV, both low‐ and high‐risk (S6, S8 and S10).

**Table 2:** Description of the strategies

Strategy	HCV screening frequency	Risk reduction	Remark
S1	Every year	–	Current screening practices
S2	Every 6 months	–	Current screening recommendations [28]
S3	Every 6 months (low‐risk) Every 3 months (high‐risk)	–	Intensive 3‐month screening for the high‐risk population (i.e. chemsex practices)
S4	Every 3 months	–	Intensive 3‐month screening for all
S5	Every year	20% (high‐risk)	S1 + risk reduction (high‐risk)
S6	Every year	20% (all)	S1 + risk reduction (all)
S7	Every 6 months	20% (high‐risk)	S2 + risk reduction (high‐risk)
S8	Every 6 months	20% (all)	S2 + risk reduction (all)
S9	Every 6 months (low‐risk) Every 3 months (high‐risk)	20% (high‐risk)	S3 + risk reduction (high‐risk)
S10	Every 3 months	20% (all)	S4 + risk reduction (all)

Note: Risk reduction strategies targeted to the high‐risk population, that is chemsex practices (S5, S7 and S9), corresponded to a 20% decrease in the relative risk of HCV transmission for the high‐risk group compared to the low‐risk group. Risk reduction strategies for all MSM living with HIV (S6, S8 and S10) corresponded to a 20% decrease in the overall transmission rate.

We considered a theoretical risk reduction intervention based on educational counselling sessions that may result in a 20% risk reduction in HCV transmission at the population level. Since MSM living with HIV are usually followed‐up yearly at the hospital, we defined a patient‐centred risk reduction counselling intervention, to be delivered by hospital medical staff alongside annual consultation. We hypothesized that this would reduce HCV transmission at the population level by 20% [16, 17, 29], including those not in care. We assumed that counselling sessions lasted 1 hour during the first year of intervention (i.e. 2021) and 30 minutes thereafter. The 20% risk reduction target was attained progressively, starting at 0% and increasing linearly over the first 12 months, then sustained due to repeated annual counselling (i.e. no subsequent change in risk practices).

### 2.5 Costs

We included direct medical lifetime costs associated with HCV screening, that is testing (serological and PCR test) and diagnosis (initial check‐up at HCV diagnosis), and HCV disease treatment and care. HCV costs stratified by disease stage were obtained from a study on healthcare consumption for chronic hepatitis C in France undertaken in 2010 [14]. Costs of HCV antiviral treatment were €24,935 or €25,750 for a 12‐week DAA cure, with or without ribavirin. Given that our strategies are assumed to result in survival gains and that MSM living with HIV are mostly engaged in HIV care, we also included HIV healthcare costs (€17,529 per year) [30, 31]. Staff costs for counselling were estimated from the hourly wage for hospital nurses (€28). Supplementary Material S4 and Table S3 provide more details on costs.

### 2.6 Health‐related quality‐of‐life

Estimates of health‐related quality‐of‐life for individuals with HIV‐HCV co‐infection are scarce [32]. We, therefore, treated such losses as the sum of losses attributable to each condition alone (Table S4). We obtained health utility estimates for chronic hepatitis C from a 2014 to 2015 cross‐sectional study of individuals with HCV mono‐infection in France using the EuroQol‐5D [33]. Estimates for cirrhosis complications were obtained from the Adelphi study [34]. Using a health utility estimate of 0.94 among individuals living with asymptomatic HIV from a meta‐analysis published in 2002 [35], we assumed that HIV infection reduces the quality‐of‐life by 0.06 points. Health utilities were left unchanged during DAA treatment. We assumed that persons whose HCV infection was cleared to fibrosis stage F3 returned to the health utility of persons with HIV mono‐infection (i.e. 0.94). Furthermore, health utility was increased after SVR for F4, as observed in Adelphi [34].

### 2.7 Model outcomes

The main outcome was the incremental cost‐effectiveness ratio (ICER), measured in euros (€) per QALY gained. A strategy's ICER was defined as its additional cost divided by its additional QALY, when compared to the next‐least‐expensive strategy. In keeping with accepted practice, strategies were deemed to be dominated and excluded from ICER calculations if they resulted in higher costs but less (or equal) benefit, or had a higher ICER than a more effective strategy [36]. The French Health Authority has elected not to establish a willingness‐to‐pay (WTP) threshold to define a “cost‐effective” intervention [18]. Accordingly, we report our results in terms of the greatest possible WTP for which an intervention might be considered cost‐effective. As a complementary outcome, we report the number of infections and cost per infection averted.

### 2.8 Sensitivity analyses

We performed one‐way deterministic sensitivity analyses to assess the robustness of our cost‐effectiveness results in the face of input data uncertainty. Uncertainty intervals were either obtained from the literature or set to plausible ranges (Table S1). We considered risk reduction levels of 15% and 10% instead of 20%. Although recent studies assessing the efficacy of DAA therapy among individuals co‐infected with HIV in real‐world settings indicate SVR rates of 95% or less [37], we explored the impact of assuming a 98% SVR rate from 2019 for those treated in disease stages preceding cirrhosis. We performed an analysis with assortative mixing. We also conducted a probabilistic sensitivity analysis (PSA) (Table S5), sampling 25,000 parameter sets and generating a cost‐effectiveness acceptability curve.

## 3 RESULTS

### 3.1 Main analysis

Strategies were sorted by increasing costs (Table 3). Under reference scenario (S1, current yearly HCV screening, no risk reduction), average lifetime costs were €351,646, mainly due to HIV care (€349,747). It also resulted in a QALY of 18.989 years. Of the 3320 new HCV infections (primary infections and reinfections) from 2021 to 2065, 484 were reinfections following a previous SVR. Although it was the least effective strategy, S1 was not the cheapest. Indeed, a 20% risk reduction intervention targeted to the high‐risk group combined with yearly screening (S5) conferred 0.003 additional QALYs (18.992) and prevented 737 new HCV infections compared to S1 (including 156 reinfections), while decreasing overall lifetime costs to €351,491.

**Table 3:** Cost‐effectiveness analysis of the different strategies

Strategy	Lifetime costs (€)	Life‐expectancy (LYs)	Quality‐adjusted life expectancy (QALYs)	Number of infections (reinfections after SVR)	ICER (€/LY)	ICER (€/QALY)
S5 = screening every year; risk reduction (high‐risk)	351,491	20.2405	18.9921	2583 (328)	–	–
S6 = screening every year; risk reduction (all)	351,605	20.2405	18.9932	2284 (303)	SD	ED
S1 = screening every year (current practices)	351,646	20.2403	18.9893	3320 (484)	SD	SD
S7 = screening every 6 months; risk reduction (high‐risk)	351,712	20.2406	18.9957	2185 (271)	2,210,000	61,389
S2 = screening every 6 months (current guidelines)	351,807	20.2404	18.9942	2701 (378)	SD	SD
S8 = screening every 6 months; risk reduction (all)	351,847	20.2406	18.9963	1966 (256)	SD	225,000
S9 = screening every 3 months (high‐risk); screening every 6 months (low‐risk); risk reduction (high‐risk)	351,924	20.2406	18.9962	2120 (262)	SD	SD
S3 = screening every 3 months (high‐risk); screening every 6 months (low‐risk)	352,013	20.2405	18.9949	2597 (362)	SD	SD
S4 = screening every 3 months	352,406	20.2405	18.9953	2550 (354)	SD	SD
S10 = screening every 3 months; risk reduction (all)	352,456	20.2406	18.9971	1882 (244)	SD	761,250

Note: The strategies were sorted by increasing costs. Incremental costs, incremental QALYs and ICERs were compared to the previous relevant strategy (i.e. not dominated). Note that lifetime costs, LYs and QALYs are presented on average per person, while the number of infections (and reinfections after SVR) is cumulative for the whole cohort throughout the simulation between 2021 and 2065.

Abbreviations: ED, extendedly dominated (at least one more expensive strategy has a lower ICER); ICER, incremental cost‐effectiveness ratio; QALY, quality‐adjusted life‐expectancy; SD, strongly dominated (more expensive and less or equally effective); SVR, sustained virological response.

**Figure 1:**
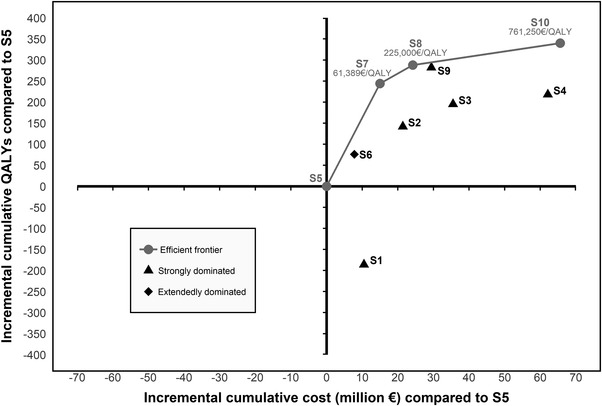
Efficiency curve. The graph plots the incremental discounted QALYs (y‐axis) and incremental discounted lifetime costs (x‐axis) for each strategy, compared to a fixed comparator (S5, yearly screening and risk reduction for the high‐risk group). The solid line represents the cost‐effectiveness frontier, that is strategies that are potentially economically efficient depending on one's willingness‐to‐pay per QALY gained. For convenience in reading the figure, we represented the cumulative QALYs and costs (in million €) instead of lifetime costs and QALYs. Abbreviation: QALY, quality‐adjusted life‐expectancy.

Compared to S5, increasing screening frequency every 6 months (S7) led to higher costs and QALYs with an ICER of €61,389/QALY. It also prevented 398 new HCV infections compared to S5, yielding a cost per infection averted of €37,790 (Table S6). From S7, the two subsequent non‐dominated strategies (S8, extending risk reduction to the low‐risk group; S10, intensifying screening every 3 months for all) yielded much higher ICERs (> €200,000/QALY), while preventing an additional 219–303 new HCV infections. All strategies without risk reduction intervention (S1–S4) were strictly dominated, that is more expensive and less effective than some other strategy, as were strategies targeting the high‐risk group for intensive screening, that is every 3 months (S3, without risk reduction; or S9, with risk reduction). Figure 1 shows the efficiency frontier for the analysis on €/QALY gained. Any strategy on the efficiency frontier is labelled “efficient” (S5, S7, S8 and S10); strategies not on the frontier are labelled “dominated” (S1, S2, S3, S4 and S6).

### 3.2 Sensitivity analyses

**Figure 2:**
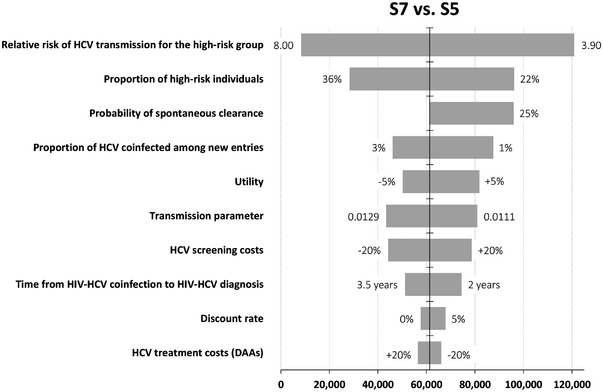
Univariate sensitivity analysis performed on incremental cost‐effectiveness ratio of strategy S7 compared to strategy S5. We compared strategy S7 (6‐month screening combined with risk reduction for the high‐risk group) with strategy S5 (yearly screening combined with risk reduction for the high‐risk group). The tornado diagram summarizes the results of a series of one‐way, deterministic sensitivity analyses to explore the robustness of the ICER per QALY gained in the face of uncertainty in the input parameters. The bars represent the range in ICER if these parameters varied across their plausible ranges. The top bar, for example, denotes the range of the ICER when we varied the relative risk of HCV transmission for the high‐risk group from its lower bound (3.90) to its upper bound (8.00). The vertical line in the middle denotes the base case ICER per QALY (€61,389). The horizontal bars were sorted according to the magnitude of variation of the ICER.

Across broad variation in the underlying parameters, S5 and S7 remained on the efficient frontier, while intermediate strategies S2, S3, S4 and S6 remained dominated. Strategies S8 and S10 also remained on the frontier but produced ICERs > €200,000/QALY, exceeding what most observers would deem acceptable. Consequently, we focused our sensitivity analysis on the choice between S5 and S7, the two most policy‐relevant strategies. Figure 2 presents the 10 parameters that produced the greatest variation in the ICER when comparing S7 and S5.

The ICER strongly depended on the relative risk of HCV transmission for the high‐risk group compared with the low‐risk group (ranging from €8378 to €120,909/QALY). Our results were also sensitive to the proportion of MSM in the high‐risk group and the probability of spontaneous clearance; a proportion of 22% in the high‐risk group and 25% of spontaneous clearance during acute infection both yielded an ICER of €96,000/QALY. S5 remained the cheapest strategy and was cost‐saving compared with current practices even when the efficacy of risk reduction interventions was set to 10%. In this case, the ICER was €47,317/QALY for S7 compared to S5 (Table S7). Finally, S7 was less cost‐effective at higher SVR rates and with assortative mixing, with ICERs of €77,338/QALY (Table S8) and €96,667/QALY compared to S5 (Table S9), respectively.

**Figure 3:**
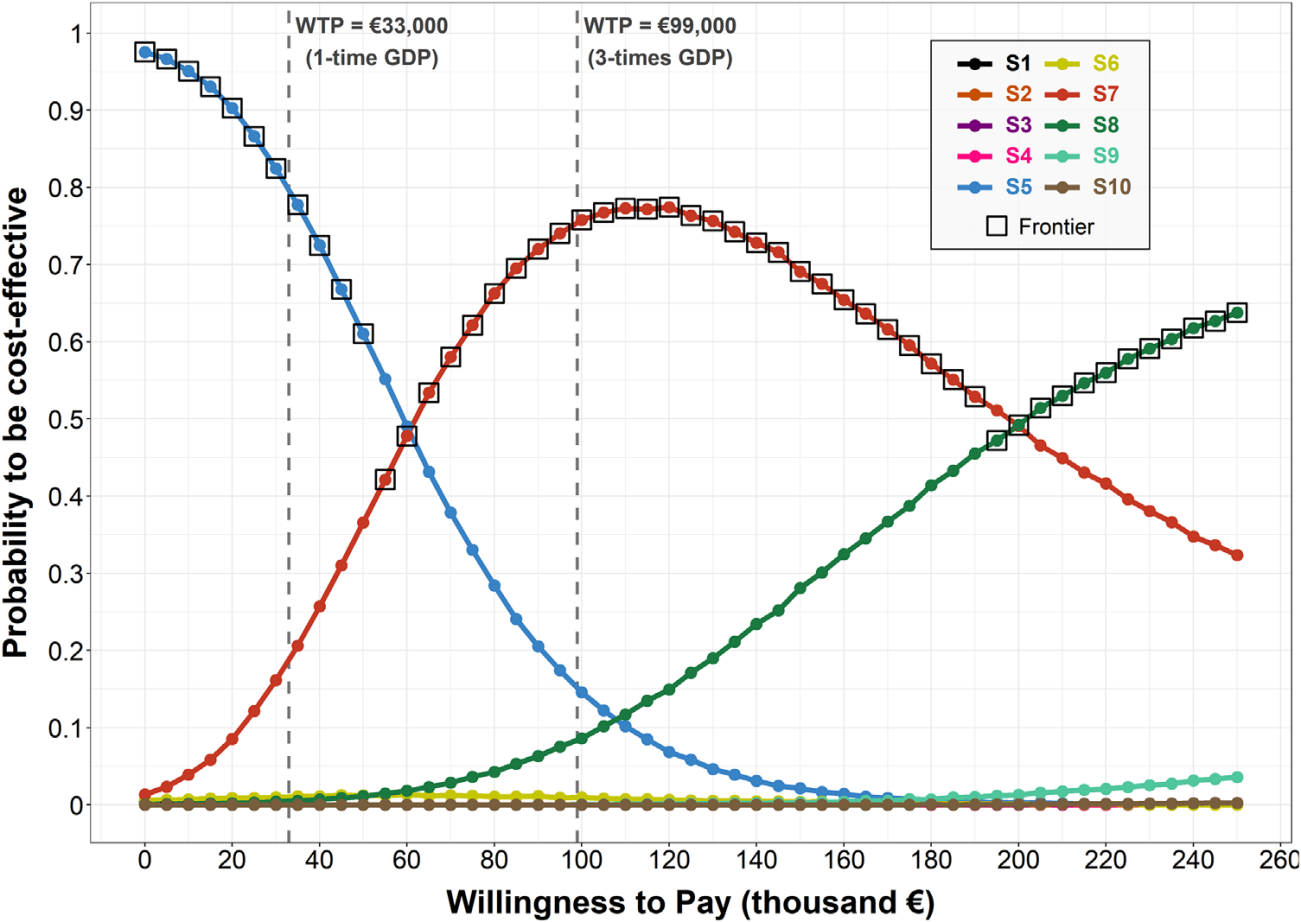
Results from the probabilistic sensitivity analysis. The cost‐effectiveness acceptability curve depicts the probability that a given strategy is cost‐effective (defined as achieving the maximum net monetary benefit [NMB]) as a function of the WTP threshold across all simulations of the probabilistic sensitivity analysis (PSA). The cost‐effectiveness acceptability frontier, represented by black squares, displays the probability of the optimal strategy (defined as maximizing the expected benefit on average across all samples of the PSA at each WTP) to be cost‐effective. In a few cases, strategies with the highest probability of being cost‐effective did not necessarily correspond with the optimal strategy, defined as having the highest expected net benefit in average, as shown by the cost‐effectiveness acceptability frontier. For example, at a WTP threshold of €55,000/QALY, S7 had the highest expected net benefit (i.e. optimal strategy), but a slightly lower probability of being cost‐effective than S5. Abbreviations: GDP, gross domestic product; WTP, willingness‐to‐pay.

In PSA, at a WTP of €33,000/QALY (roughly the French Gross Domestic Product [GDP]), S5 was cost‐effective in 80% of simulations, and S7 in 17% of simulations. S7 was cost‐effective with a 75% probability at a WTP of roughly three times the GDP (i.e. €99,000) and remained the strategy with the highest probability of being cost‐effective when considering a WTP up to €200,000/QALY (Figure 3).

## 4 DISCUSSION

This study aimed to assess the cost‐effectiveness of HCV screening and risk reduction strategies among MSM living with HIV in France. Two key points may be drawn from this analysis. Firstly, risk reduction interventions have a strong potential to be efficient, with a 20% risk reduction intervention in the high‐risk group being cost‐saving compared with current practices. The efficiency of intensified HCV screening alone appears low. Secondly, combining a 20% risk reduction intervention with a 6‐month HCV screening might be cost‐effective compared with yearly screening (ICER = €61,389/QALY). While the French Health Authority has taken no official position with regard to a national WTP threshold, the *per capita* GDP (€33,270 in 2019 [38]) offers a point of departure to interpret our results, as others have previously done [39, 40]. Using this basis of comparison, we might define a programme to be “cost‐effective” if its ICER is less than three times the *per capita* GDP (€99,810), and “very cost‐effective” if its ICER falls below the GDP (€33,270). In our analysis, screening every 6 months combined with risk reduction resulted in an ICER of about two times the French GDP. This strategy would not be considered as cost‐effective with a WTP of one time the GDP, but would be regarded as cost‐effective for a WTP threshold between €60,000 and €200,000 per QALY gained. Additionally, combining a 20% risk reduction intervention with a 6‐month HCV screening provided a cost per infection averted of €37,790 compared with yearly screening.

Although MSM engaged in high‐risk practices drive the HCV epidemic, intensified screening every 3 months in this population (while screening the low‐risk group every 6 months) was not cost‐effective, even if we assumed no additional cost to identify high‐risk persons. This suggests that the health gains offered by more frequent screening would not justify the additional costs.

Overall, HCV screening strategies had no impact on the number of LYs gained and a limited impact on the number of QALYs. Our results highlight that routine screening every year is an already favourable situation, especially when a high proportion of MSM living with HIV are engaged in care and rapidly treated once diagnosed. Moreover, since chronic HCV infection can progress slowly (20–30 years to cirrhosis [41, 42]), the marginal benefit of screening more than once a year may be small in terms of morbidity and mortality. Although our model allows for progression to advanced disease, most persons with HCV who are already under HIV care progress no further than acute infection or fibrosis stage F0. This is particularly pertinent in France, where heightened awareness has led to decreasing HCV incidence, a low prevalence (3.62% in 2014) and comparatively few MSM in the most severe stages of HCV infection.

A few recently published studies used dynamic compartmental modelling to evaluate the cost‐effectiveness of different HCV screening and treatment interventions among MSM living with HIV in the Netherlands [43, 44], or among all MSM (not only those living with HIV) in the United Kingdom [45]. Popping et al. reported minimal QALY benefit from frequent HCV testing in Dutch MSM living with HIV [44]. However, the authors showed that a simplified HCV screening strategy using lower‐cost but equally effective antigen tests (6‐monthly, targeted to MSM living with HIV with a previously known HCV infection) was cost‐saving compared with current monitoring and testing strategies (based on PCR tests in case of elevated alanine aminotransferase or positive serological test). Our study contributes to the existing literature by simultaneously assessing both testing and risk reduction strategies and by identifying efficient combinations of both interventions among French MSM living with HIV.

This study has limitations. Firstly, uncertainties persist regarding the input data of the model. However, risk reduction targeted to the high‐risk group remained cost‐saving in all sensitivity analyses. Secondly, our model is specific to France where favourable circumstances prevail regarding HCV screening and treatment among MSM living with HIV. It might be useful to explore the generalizability of our findings to other settings. However, there is reason to believe that our conclusions regarding the efficiency of risk reduction strategies will apply to other high‐income settings with similar HCV epidemics among MSM (i.e. decreasing incidence [46, 47, 48]). Engagement in chemsex and other high‐risk sexual practices substantially contributes to HCV transmission among MSM living with HIV in all settings, and a number of modelling studies have shown that micro‐elimination cannot be achieved without implementing risk reduction interventions [15, 49, 50]. Thirdly, our compartmental model design and homogeneous mixing assumption may be less appropriate for evaluating strategies targeted to specific populations, compared to an individual‐based simulation and/or an assortative mixing preference between risk groups [51]. HCV screening every 6 months proved less cost‐effective when we assumed assortative mixing, mostly because increased screening added less value to risk reduction. We may also have over‐simplified the reality of risk‐reduction programme delivery. While we assumed counselling sessions were dispensed by the medical staff for practicality, peer‐counselling could be more relevant in this situation. There is, unfortunately, a paucity of data regarding risk reduction interventions aimed to reduce HCV transmission among MSM engaged in high‐risk practices. Nevertheless, our aim was to provide valuable information on whether such strategies could be cost‐effective. In addition, we assumed that all HCV transmission in MSM living with HIV in France was attributable to contacts within this population. Previous studies have described both an international network of HCV transmission among MSM living with HIV [52, 53] and HCV infections among MSM without HIV infection receiving HIV pre‐exposure prophylaxis (PrEP) [9, 54]. However, we found no estimate of what proportion of HCV incidence among MSM living with HIV in France is attributable to contact with these other populations. It also remains unclear whether and how PrEP users could influence the dynamics of HCV infections among MSM living with HIV. For instance, PrEP prescription requires regular follow‐up, providing more opportunities to be tested for HCV, which could contribute to reducing HCV incidence among both populations. Another modelling assumption was that all MSM living with HIV were equally likely to be diagnosed. Future research might consider heterogeneity in HIV screening rates among different MSM subgroups and the potential costs and benefits of interventions targeted to persons facing unusually high barriers to care. Finally, we cannot entirely rule out that current practices already tend towards 6‐month HCV screening among MSM living with HIV in France. However, our results suggest that this might possibly be an optimal screening frequency.

## 5 CONCLUSIONS

In conclusion, the risk reduction was a critical intervention in our analysis, indicating the high value of targeting chemsex and other high‐risk practices among MSM living with HIV in France. Thus, depending on policymakers’ WTP, screening MSM living with HIV every 6 months for HCV might be considered as cost‐effective, in combination with risk reduction.

## COMPETING INTERESTS

KC has received honoraria for presentations from Gilead outside of this work. VS reports lecture fees from Gilead, Janssen and Viiv outside the submitted work. JG reports personal fees from Merck, grants and personal fees from ViiV healthcare, grants and personal fees from Gilead Sciences, personal fees from Roche, personal fees from AstraZeneca, and personal fees from Janssen, outside the submitted work. SDB has received consultancy fees from Intercept. The remaining authors have nothing to disclose.

## AUTHORS’ CONTRIBUTIONS

MC: Study concept and design, methodology, acquisition of data, formal analysis and interpretation of results, drafting of the manuscript, critical revision of the manuscript for important intellectual content, obtained funding; AC: Study concept and design, methodology, interpretation of results, drafting of the manuscript, critical revision of the manuscript for important intellectual content, study supervision, obtained funding; KC: Methodology, critical revision of the manuscript for important intellectual content; VS: Interpretation of results, critical revision of the manuscript for important intellectual content; AV: Critical revision of the manuscript for important intellectual content; JG: Critical revision of the manuscript for important intellectual content; YY: Study concept and design, critical revision of the manuscript for important intellectual content; ADP: Methodology, interpretation of results, critical revision of the manuscript for important intellectual content; SDB: Study concept and design, methodology, interpretation of results, drafting of the manuscript, critical revision of the manuscript for important intellectual content, study supervision, obtained funding. All authors approved the final version of the manuscript, and agree to be accountable for all aspects of the work. All authors have given final approval for the submission to *Journal of the International AIDS Society*.

## FUNDING

This work was supported by the French Agency for Research on AIDS and Viral Hepatitis (ANRS) [grant number 95031]. The ANRS had no role in the study design, data collection and analysis, decision to publish or preparation of the manuscript.

## Supporting information


**Table S1**. Model parameters values
**Table S2**. Assumptions for HCV treatment uptake evolution among MSM living with HIV in France from 2014 to 2019
**Table S3**. Annual mean costs attributable to chronic hepatitis C: ambulatory costs (never treated and after HCV treatment failure) and hospitalization costs (no death and in‐hospital death) [9]
**Table S4**. Health‐related utilities
**Table S5**. Distributions used in the probabilistic sensitivity analysis
**Table S6**. Description of costs, number of infections, and cost per infection averted for nondominated strategies on the efficiency frontier
**Table S7**. Sensitivity analysis decreasing the efficacy of risk reduction strategies to 15% and 10%
**Table S8**. Sensitivity analysis assuming a 98% SVR rate from 2019 for those treated before cirrhosis (i.e., acute infection and fibrosis stages F0, F1, F2, F3)
**Table S9**. Sensitivity analysis assuming assortative mixing
**Figure S1**. Flow diagrams of the HCV transmission and progression model.

## Data Availability

Most of the data that support the findings of this study are available from the cited references and supplementary material. PREVAGAY 2015 data are available from the authors upon reasonable request and additional approval from Santé Publique France. Other data are available from the authors upon reasonable request. Anyone can submit a research project to the ANRS CO4‐FHDH scientific committee and obtain access to the data after approval by the scientific committee. Applicants should use a standardized form available on the ANRS CO4‐FHDH website (https://anrs‐co4.fhdh.fr) to describe the context and objectives of the study. The scientific committee reviews the submitted projects twice a year. For successful applicants with adequate statistical expertise, the data can be transferred with French data protection agency CNIL approval; otherwise, the ANRS CO4 FHDH statistical centre analyses the data cooperatively with the applicant. Anyone can submit a research project to Santé Publique France and obtain access to the routine HIV surveillance data after approval by the scientific committee, by writing to ANSP-DMI-VIC@santepubliquefrance.fr.
