# Supplementary material for: Cost‐effectiveness of hepatitis C virus test‐and‐treat and risk reduction strategies among men who have sex with men living with HIV in France
Source: J Int AIDS Soc. 2022 Nov 30;25(12):e26035. doi: 10.1002/jia2.26035 (PMC9712801; doi:10.1002/jia2.26035)
Supplement: Supplementary file 1 — Table S1. Model parameters values Table S2. Assumptions for HCV treatment uptake evolution among MSM living with HIV in France from 2014 to 2019 Table S3. Annual mean costs attributable to chronic hepatitis C: ambulatory costs (never treated and after HCV treatment failure) and hospitalization costs (no death and in‐hospital death) [9] Table S4. Health‐related utilities Table S5. Distributions used in the probabilistic sensitivity analysis Table S6. Description of costs, number of infections, and cost per infection averted for nondominated strategies on the efficiency frontier Table S7. Sensitivity analysis decreasing the efficacy of risk reduction strategies to 15% and 10% Table S8. Sensitivity analysis assuming a 98% SVR rate from 2019 for those treated before cirrhosis (i.e., acute infection and fibrosis stages F0, F1, F2, F3) Table S9. Sensitivity analysis assuming assortative mixing Figure S1. Flow diagrams of the HCV transmission and progression model. [file JIA2-25-e26035-s001.docx]

Supplementary Information

Cost-effectiveness of hepatitis C virus test-and-treat and risk reduction strategies among men who have sex with men living with HIV in France

Mathieu Castry^1^, Anthony Cousien^1^, Karen Champenois^1^, Virginie Supervie^2^, Annie Velter^3^, Jade Ghosn^1,4^, Yazdan Yazdanpanah^1,4^, A. David Paltiel^5^, Sylvie Deuffic-Burban^1^

^1^Université de Paris, Inserm, IAME, F-75006 Paris, France

^2^Sorbonne Université, Inserm, Institut Pierre Louis d’Épidémiologie et de Santé Publique, Paris, France

^3^Santé Publique France, Saint-Maurice, France

^4^Service de maladies Infectieuses et tropicales, Hôpital Bichat Claude Bernard, Paris, France

^5^Yale School of Public Health, New Haven CT, USA

Table of contents

[S1. Model structure 3](#_Toc114744806)

[1.1. Risk stratification 3](#_Toc114744808)

[1.2. HCV infection, HCV and HIV cascades of care 3](#_Toc114744809)

[1.3. Hepatitis C disease progression model 4](#_Toc114744810)

[S2. Difference equations 5](#_Toc114744811)

[S3. Parameters estimations and model calibration 10](#_Toc114744812)

[S4. Cost data 11](#_Toc114744813)

[S5. Sensitivity analysis assuming assortative mixing 12](#_Toc114744814)

[S6. Supplementary figures and tables 13](#_Toc114744815)

[S7. References 28](#_Toc114744816)

Supplementary figures

[**Figure S1.** Flow diagrams of the HCV transmission and progression model. 13](#_Toc91785038)

Supplementary tables

[**Table S1.** Model parameters values 14](#_Toc114744878)

[**Table S2.** Assumptions for HCV treatment uptake evolution among MSM living with HIV in France from 2014 to 2019 17](#_Toc114744879)

[**Table S3.** Annual mean costs attributable to chronic hepatitis C: ambulatory costs (never treated and after HCV treatment failure) and hospitalization costs (no death and in-hospital death) 18](#_Toc114744880)

[**Table S4.** Health-related utilities 19](#_Toc114744881)

[**Table S5.** Distributions used in the probabilistic sensitivity analysis 20](#_Toc114744882)

[**Table S6.** Description of costs, number of infections, and cost per infection averted for non-dominated strategies on the efficiency frontier 24](#_Toc114744883)

[**Table S7.** Sensitivity analysis decreasing the efficacy of risk reduction strategies to 15% and 10% 25](#_Toc114744884)

[**Table S8.** Sensitivity analysis assuming a 98% SVR rate from 2019 for those treated before cirrhosis (i.e., acute infection and fibrosis stages F0, F1, F2, F3) 26](#_Toc114744885)

[**Table S9.** Sensitivity analysis assuming assortative mixing 27](#_Toc114744886)

# **S1. Model structure**

A deterministic, discrete-time, compartmental model describing the hepatitis C epidemic among men who have sex with men (MSM) was developed using R programming language [1]. The complete flow diagram of the model structure is presented in **Figure S1**.


## **Risk stratification**

We stratified the population of MSM living with HIV into two risk groups (low and high) to account for heterogeneity in sexual and drug-related behaviors and risk of acquiring HCV, using chemsex practices as a proxy. We used the subscript $r \epsilon\{l,h\}$ ($l$ stands for low-risk and $h$ stands for high-risk) to denote these two populations. It should be noted that we assumed no transition between risk groups, so that the proportion of high-risk MSM remained stable throughout the simulation.

## **HCV infection, HCV and HIV cascades of care**

The flow diagram describing the HCV transmission model is shown in **Figure S1.A**. Each new individual entered the model as a newly HIV-infected and undiagnosed MSM, either HCV uninfected (ND_X) or already HCV coinfected (ND_ND). Entry in the model was the yearly number of new HIV infections ($\pi$), i.e. HIV incidence among MSM in France. We assumed that a proportion entered in the model with HCV coinfection ($\pi'$). Those who were HCV-uninfected (ND_X) could become acutely infected with a force of infection which depended on their sexual risk behaviour ($\lambda_{r}$) and became undiagnosed for both infections (ND_ND). Otherwise, if not HCV-infected, they could become diagnosed for their HIV infection at a rate $\tau a$. Once diagnosed for HIV they could be acutely infected with HCV (D_ND) with the same force of infection $\lambda_{r}$. If not, they could be enrolled in care (LC_X) at a rate $\phi a$. Once linked to care they also had a risk to become HCV-infected (LC_ND). Moreover, we hypothesized that HIV-diagnosed but HCV-undiagnosed individuals (D_ND) entered in HIV care first (LC_ND), before entering in HCV care (LC_LC) at a rate $\phi b$. More importantly, $\phi b$ was the parameter used for screening strategies (we considered HCV screening as a proxy for entry into HCV care among MSM already in HIV care).

Those undiagnosed for both infections (ND_ND) could be diagnosed for HIV and HCV at a rate $\tau b$, the underlying assumption being that if a coinfected MSM was diagnosed for one infection (either HIV or HCV) then he was necessarily diagnosed for the other one (i.e. with a HIV-HCV screening). They could subsequently enter in care for both infections (LC_LC) at a rate $\phi c$, and be treated at a rate $\alpha$. Treatment had a fixed duration $T_{t}$ and could lead to sustained virological response (SVR) with a probability $\mathrm{pSVR}$. Those who achieved SVR between acute infection and fibrosis stage F3 cleared the virus and became susceptible to re-infection; i.e. they returned to the susceptible state (LC_X) and could be re-infected in the same manner as MSM who had never been infected with HCV. Specifically, we divided the following compartments to distinguish reinfections from primary infections: D_X, LC_X, D_ND, D_D, LC_ND and LC_LC. If they achieved SVR from fibrosis stage F4 we assumed they could progress to DC and HCC (LC_ST), although at reduced risk compared with those who did not achieve SVR (LC_FT). We assumed MSM could not escape this state (LC_FT), since we did not include the possibility of retreatment for non-responders. However, a MSM could be treated again in the case of re-infection after SVR. Once linked to care, individuals could be loss to follow-up at rate $\varphi$ and came back in a diagnosed, non-linked to care state.

## **Hepatitis C disease progression model**

The model also tracked the HCV disease progression, as displayed in **Figure S1.B**. We used the subscript $h$ to distinguish the stages of the disease. The first part of the model describes the acute infection phase. During that period a proportion ($\mathrm{psc}$) of individuals spontaneously cleared their acute infection after a duration of time ($\mathrm{Ta}$) and returned to a susceptible compartment. Those who did not spontaneously clear their acute infection ($1-psc$) progressed to chronic infection. Then the model described the fibrosis progression. METAVIR scores are used to classify stages of liver disease: from F0 (no fibrosis) to F4 (compensated cirrhosis). Chronically infected individuals at fibrosis stage F4 could then progress to decompensated cirrhosis (DC) or develop hepatocellular carcinoma (HCC). Individuals in DC stage could also develop HCC. Patients in liver decompensation or HCC could be considered for liver transplantation (LT). Finally, a specific HCV-related risk of mortality was applied to the most severe stages of chronic hepatitis C (DC, HCC, or LT).

# **S2. Difference equations**

For simplicity, we used a notation with subscripts for risk groups (with $\text{r ϵ \{l,h\}}$ corresponding to low-risk and high-risk group) and disease progression (with $\text{h }\text{ϵ \{0,1,2,3,4,5,6,7,8\}}$). When possible, we summarized the equations for several values of disease progression (e.g. when $\text{h}$ = 2,3,4, corresponding to fibrosis stages F1, F2, F3). We adopted the following system of difference equations to model HCV transmission among MSM living with HIV in France:

**Susceptible compartments**

$$\boldsymbol{ND\_X}_{\mathbf{r}}^{\mathbf{t+1}}={ND\_X}_{r}^{t}+\pi_{r}+psc\left( \frac{1}{\mathrm{Ta}} \right){ND\_ND}_{r,0}^{t}-(\tau_{a}+\mu+\lambda_{r}){ND\_X}_{r}^{t}$$

${\mathbf{D}\boldsymbol{\_}\mathbf{X}}_{\mathbf{r}}^{\mathbf{t}\mathbf{+}\mathbf{1}}={D\_X}_{r}^{t}+\tau_{a}\left( {ND\_X}_{r}^{t} \right)+\varphi\left( {LC\_X}_{r}^{t} \right)+psc\left( \frac{1}{\mathrm{Ta}} \right)\left( {D\_ND}_{r,0}^{t}+{D\_D}_{r,0}^{t} \right)-(\phi_{a}+\mu+\lambda_{r}){D\_X}_{r}^{t}$

${\mathbf{LC}\boldsymbol{\_}\mathbf{X}}_{\mathbf{r}}^{\mathbf{t}\mathbf{+}\mathbf{1}}={LC\_X}_{r}^{t}+\phi_{a}\left( {D\_X}_{r}^{t} \right)+\left( p_{\mathrm{RVS}}\times\frac{1}{\mathrm{Tt}} \right){LC\_TR}_{r,h\in\left[ 0;4 \right]}^{t}+psc\left( \frac{1}{\mathrm{Ta}} \right)\left( {LC\_ND}_{r,0}^{t}+{LC\_LC}_{r,0}^{t} \right)-(\varphi+\mu+\lambda_{r}){LC\_X}_{r}^{t}$

**HIV-HCV undiagnosed compartments**

$\boldsymbol{ND\_ND}_{\mathbf{r,0}}^{\mathbf{t+1}}={ND\_ND}_{r,0}^{t}+{\pi^{'}}_{r,0}+\lambda_{r}\left( {ND\_X}_{r}^{t} \right)-\left( \tau b+\mu+\frac{1}{\mathrm{Ta}} \right){ND\_ND}_{r,0}^{t}$

$\boldsymbol{ND\_ND}_{\mathbf{r,1}}^{\mathbf{t+1}}={ND\_ND}_{r,1}^{t}+{\pi^{'}}_{r,1}+\left( 1-psc \right)\left( \frac{1}{\mathrm{Ta}} \right){ND\_ND}_{r,0}^{t}-\left( \tau b+\mu+\rho_{12} \right){ND\_ND}_{r,1}^{t}$

$for h\in\left[ 2,3,4 \right], \boldsymbol{ND\_ND}_{\mathbf{r,h}}^{\mathbf{t+1}}={ND\_ND}_{r,h}^{t}+{\pi^{'}}_{r,h}+\rho_{\left( h-1 \right)h}\left( {ND\_ND}_{r,h-1}^{t} \right)-\left( \tau b+\mu+\rho_{h\left( h+1 \right)} \right){ND\_ND}_{r,h}^{t}$

$\boldsymbol{ND\_ND}_{\mathbf{r,5}}^{\mathbf{t+1}}={ND\_ND}_{r,5}^{t}+{\pi^{'}}_{r,5}+\rho_{45}\left( {ND\_ND}_{r,4}^{t} \right)-\left( \tau b+\mu+\rho_{56}+\rho_{57} \right){ND\_ND}_{r,5}^{t}$

$\boldsymbol{ND\_ND}_{\mathbf{r,6}}^{\mathbf{t+1}}={ND\_ND}_{r,6}^{t}+{\pi^{'}}_{r,6}+\rho_{56}\left( {ND\_ND}_{r,5}^{t} \right)-\left( \tau b+\mu+\rho_{67}+\rho_{68}+\rho_{69} \right){ND\_ND}_{r,6}^{t}$

$\boldsymbol{ND\_ND}_{\mathbf{r,7}}^{\mathbf{t+1}}={ND\_ND}_{r,7}^{t}+{\pi^{'}}_{r,7}+\rho_{57}\left( {ND\_ND}_{r,5}^{t} \right)+\rho_{67}\left( {ND\_ND}_{r,6}^{t} \right)-\left( \tau b+\mu+\rho_{78}+\rho_{79} \right){ND\_ND}_{r,7}^{t}$

$\boldsymbol{ND\_ND}_{\mathbf{r,8}}^{\mathbf{t+1}}={ND\_ND}_{r,8}^{t}+{\pi^{'}}_{r,8}+\rho_{68}\left( {ND\_ND}_{r,6}^{t} \right)+\rho_{78}\left( {ND\_ND}_{r,7}^{t} \right)-\left( \tau b+\mu\right){ND\_ND}_{r,8}^{t}$

$\boldsymbol{ND\_ND}_{\mathbf{r,9}}^{\mathbf{t+1}}={ND\_ND}_{r,9}^{t}+\rho_{69}\left( {ND\_ND}_{r,6}^{t} \right)+\rho_{79}\left( {ND\_ND}_{r,7}^{t} \right)$

**HIV-HCV diagnosed compartments**

$\boldsymbol{D\_D}_{\mathbf{r,0}}^{\mathbf{t+1}}={D\_D}_{r,0}^{t}+\tau b\left( {ND\_ND}_{r,0}^{t} \right)+\varphi({LC\_LC}_{r,0}^{t})-\left( \phi c+\mu+\frac{1}{\mathrm{Ta}} \right){D\_D}_{r,0}^{t}$

$\boldsymbol{D\_D}_{\mathbf{r,1}}^{\mathbf{t+1}}={D\_D}_{r,1}^{t}+\left( 1-psc \right)\left( \frac{1}{\mathrm{Ta}} \right){D\_D}_{r,0}^{t}+\tau b({ND\_ND}_{r,1}^{t})+\varphi({LC\_LC}_{r,1}^{t})-\left( \phi c+\mu+\rho_{12} \right){D\_D}_{r,1}^{t}$

$for h\in\left[ 2,3,4 \right], \boldsymbol{D\_D}_{\mathbf{r,h}}^{\mathbf{t+1}}={D\_D}_{r,h}^{t}+\rho_{\left( h-1 \right)h}\left( {D\_D}_{r,h-1}^{t} \right)+\tau b({ND\_ND}_{r,h}^{t})+\varphi({LC\_LC}_{r,h}^{t})-\left( \phi c+\mu+\rho_{h\left( h+1 \right)} \right){D\_D}_{r,h}^{t}$

$\boldsymbol{D\_D}_{\mathbf{r,5}}^{\mathbf{t+1}}={D\_D}_{r,5}^{t}+\varphi({LC\_LC}_{r,5}^{t})+\rho_{45}\left( {D\_D}_{r,4}^{t} \right)+\tau b({ND\_ND}_{r,5}^{t})-\left( \phi c+\mu+\rho_{56}+\rho_{57} \right){D\_D}_{r,5}^{t}$

$\boldsymbol{D\_D}_{\mathbf{r,6}}^{\mathbf{t+1}}={D\_D}_{r,6}^{t}+\varphi({LC\_LC}_{r,6}^{t})+\rho_{56}\left( {D\_D}_{r,5}^{t} \right)+\tau b({ND\_ND}_{r,6}^{t})-\left( \phi c+\mu+\rho_{67}+\rho_{68}+\rho_{69} \right){D\_D}_{r,6}^{t}$

$\boldsymbol{D\_D}_{\mathbf{r,7}}^{\mathbf{t+1}}={D\_D}_{r,7}^{t}+\varphi({LC\_LC}_{r,7}^{t})+\rho_{57}\left( {D\_D}_{r,5}^{t} \right)+\rho_{67}\left( {D\_D}_{r,6}^{t} \right)+\tau b({ND\_ND}_{r,7}^{t})-\left( \phi c+\mu+\rho_{78}+\rho_{79} \right){D\_D}_{r,7}^{t}$

$\boldsymbol{D\_D}_{\mathbf{r,8}}^{\mathbf{t+1}}={D\_D}_{r,8}^{t}+\varphi({LC\_LC}_{r,8}^{t})+\rho_{68}\left( {D\_D}_{r,6}^{t} \right)+\rho_{78}\left( {D\_D}_{r,7}^{t} \right)+\tau b({ND\_ND}_{r,8}^{t})-\left( \phi c+\mu\right){D\_D}_{r,8}^{t}$

$\boldsymbol{D\_D}_{\mathbf{r,9}}^{\mathbf{t+1}}={D\_D}_{r,9}^{t}+\rho_{69}\left( {D\_D}_{r,6}^{t} \right)+\rho_{79}\left( {D\_D}_{r,7}^{t} \right)$

**HIV diagnosed, HCV undiagnosed compartments**

$\boldsymbol{D\_ND}_{\mathbf{r,0}}^{\mathbf{t+1}}={D\_ND}_{r,0}^{t}+\lambda_{r}\left( {D\_X}_{r}^{t} \right)+\varphi({LC\_ND}_{r,0}^{t})-\left( \phi a+\mu+\frac{1}{\mathrm{Ta}} \right){D\_ND}_{r,0}^{t}$

$\boldsymbol{D\_ND}_{\mathbf{r,1}}^{\mathbf{t+1}}={D\_ND}_{r,1}^{t}+\left( 1-psc \right)\left( \frac{1}{\mathrm{Ta}} \right){D\_ND}_{r,0}^{t}+\varphi({LC\_ND}_{r,1}^{t})-\left( \phi a+\mu+\rho_{12} \right){D\_ND}_{r,1}^{t}$

$for h\in\left[ 2,3,4 \right], \boldsymbol{D\_ND}_{\mathbf{r,h}}^{\mathbf{t+1}}={D\_ND}_{r,h}^{t}+\rho_{\left( h-1 \right)h}\left( {D\_ND}_{r,h-1}^{t} \right)+\varphi({LC\_ND}_{r,h}^{t})-\left( \phi a+\mu+\rho_{h\left( h+1 \right)} \right){D\_ND}_{r,h}^{t}$

$\boldsymbol{D\_ND}_{\mathbf{r,5}}^{\mathbf{t+1}}={D\_ND}_{r,5}^{t}+\rho_{45}\left( {D\_ND}_{r,4}^{t} \right)+\varphi({LC\_ND}_{r,5}^{t})-\left( \phi a+\mu+\rho_{56}+\rho_{57} \right){D\_ND}_{r,5}^{t}$

$\boldsymbol{D\_ND}_{\mathbf{r,6}}^{\mathbf{t+1}}={D\_ND}_{r,6}^{t}+\rho_{56}\left( {D\_ND}_{r,5}^{t} \right)+\varphi({LC\_ND}_{r,6}^{t})-\left( \phi a+\mu+\rho_{67}+\rho_{68}+\rho_{69} \right){D\_ND}_{r,6}^{t}$

$\boldsymbol{D\_ND}_{\mathbf{r,7}}^{\mathbf{t+1}}={D\_ND}_{r,7}^{t}+\rho_{57}\left( {D\_ND}_{r,5}^{t} \right)+\rho_{67}\left( {D\_ND}_{r,6}^{t} \right)+\varphi({LC\_ND}_{r,7}^{t})-\left( \phi a+\mu+\rho_{78}+\rho_{79} \right){D\_ND}_{r,7}^{t}$

$\boldsymbol{D\_ND}_{\mathbf{r,8}}^{\mathbf{t+1}}={D\_ND}_{r,8}^{t}+\rho_{68}\left( {D\_ND}_{r,6}^{t} \right)+\rho_{78}\left( {D\_ND}_{r,7}^{t} \right)+\varphi({LC\_ND}_{r,8}^{t})-\left( \phi a+\mu\right){D\_ND}_{r,8}^{t}$

$\boldsymbol{D\_ND}_{\mathbf{r,9}}^{\mathbf{t+1}}={D\_ND}_{r,9}^{t}+\rho_{69}\left( {D\_ND}_{r,6}^{t} \right)+\rho_{79}\left( {D\_ND}_{r,7}^{t} \right)$

**HIV linked to care, HCV undiagnosed compartments**

$\boldsymbol{LC\_ND}_{\mathbf{r,0}}^{\mathbf{t+1}}={LC\_ND}_{r,0}^{t}+\lambda_{r}\left( {LC\_X}_{r}^{t} \right)+\phi a({D\_ND}_{r,0}^{t})-\left( \varphi+\phi b+\mu+\frac{1}{\mathrm{Ta}} \right){LC\_ND}_{r,0}^{t}$

$\boldsymbol{LC\_ND}_{\mathbf{r,1}}^{\mathbf{t+1}}={LC\_ND}_{r,1}^{t}+\left( 1-psc \right)\left( \frac{1}{\mathrm{Ta}} \right){LC\_ND}_{r,0}^{t}+\phi a({D\_ND}_{r,1}^{t})-\left( \varphi+\phi b+\mu+\rho_{12} \right){LC\_ND}_{r,1}^{t}$

$for h\in\left[ 2,3,4 \right], \boldsymbol{LC\_ND}_{\mathbf{r,h}}^{\mathbf{t+1}}={LC\_ND}_{r,h}^{t}+\rho_{\left( h-1 \right)h}\left( {LC\_ND}_{r,h-1}^{t} \right)+\phi a({D\_ND}_{r,h}^{t})-\left( \varphi+\phi b+\mu+\rho_{h\left( h+1 \right)} \right){LC\_ND}_{r,h}^{t}$

$\boldsymbol{LC\_ND}_{\mathbf{r,5}}^{\mathbf{t+1}}={LC\_ND}_{r,5}^{t}+\rho_{45}\left( {LC\_ND}_{r,4}^{t} \right)+\phi a({D\_ND}_{r,5}^{t})-\left( \varphi+\phi b+\mu+\rho_{56}+\rho_{57} \right){LC\_ND}_{r,5}^{t}$

$\boldsymbol{LC\_ND}_{\mathbf{r,6}}^{\mathbf{t+1}}={LC\_ND}_{r,6}^{t}+\rho_{56}\left( {LC\_ND}_{r,5}^{t} \right)+\phi a({D\_ND}_{r,6}^{t})-\left( \varphi+\phi b+\mu+\rho_{67}+\rho_{68}+\rho_{69} \right){LC\_ND}_{r,6}^{t}$

$\boldsymbol{LC\_ND}_{\mathbf{r,7}}^{\mathbf{t+1}}={LC\_ND}_{r,7}^{t}+\rho_{57}\left( {LC\_ND}_{r,5}^{t} \right)+\rho_{67}\left( {LC\_ND}_{r,6}^{t} \right)+\phi a({D\_ND}_{r,7}^{t})-\left( \varphi+\phi b+\mu+\rho_{78}+\rho_{79} \right){LC\_ND}_{r,7}^{t}$

$\boldsymbol{LC\_ND}_{\mathbf{r,8}}^{\mathbf{t+1}}={LC\_ND}_{r,8}^{t}+\rho_{68}\left( {LC\_ND}_{r,6}^{t} \right)+\rho_{78}\left( {LC\_ND}_{r,7}^{t} \right)+\phi a({D\_ND}_{r,8}^{t})-\left( \varphi+\phi b \right){LC\_ND}_{r,8}^{t}$

$\boldsymbol{LC\_ND}_{\mathbf{r,9}}^{\mathbf{t+1}}={LC\_ND}_{r,9}^{t}+\rho_{69}\left( {LC\_ND}_{r,6}^{t} \right)+\rho_{79}\left( {LC\_ND}_{r,7}^{t} \right)$

**HIV-HCV linked to care compartments**

$\boldsymbol{LC\_LC}_{\mathbf{r,0}}^{\mathbf{t+1}}={LC\_LC}_{r,0}^{t}+\phi b({LC\_ND}_{r,0}^{t})+\phi c({D\_D}_{r,0}^{t})-\left( \varphi+\alpha+\mu+\frac{1}{\mathrm{Ta}} \right){LC\_LC}_{r,0}^{t}$

$\boldsymbol{LC\_LC}_{\mathbf{r,1}}^{\mathbf{t+1}}={LC\_LC}_{r,1}^{t}+\left( 1-psc \right)\left( \frac{1}{\mathrm{Ta}} \right){LC\_LC}_{r,0}^{t}+\phi b({LC\_ND}_{r,1}^{t})+\phi c({D\_D}_{r,1}^{t})-\left( \varphi+\alpha+\mu+\rho_{12} \right){LC\_LC}_{r,1}^{t}$

$for h\in\left[ 2,3,4 \right], \boldsymbol{LC\_LC}_{\mathbf{r,h}}^{\mathbf{t+1}}={LC\_LC}_{r,h}^{t}+\rho_{\left( h-1 \right)h}\left( {LC\_LC}_{r,h-1}^{t} \right)+\phi b({LC\_ND}_{r,h}^{t})+\phi c({D\_D}_{r,h}^{t})-\left( \varphi+\alpha+\mu+\rho_{h\left( h+1 \right)} \right){LC\_LC}_{r,h}^{t}$

$\boldsymbol{LC\_LC}_{\mathbf{r,5}}^{\mathbf{t+1}}={LC\_LC}_{r,5}^{t}+\rho_{45}\left( {LC\_LC}_{r,4}^{t} \right)+\phi b({LC\_ND}_{r,5}^{t})+\phi c({D\_D}_{r,5}^{t})-\left( \varphi+\alpha+\mu+\rho_{56}+\rho_{57} \right){LC\_LC}_{r,5}^{t}$

$\boldsymbol{LC\_LC}_{\mathbf{r,6}}^{\mathbf{t+1}}={LC\_LC}_{r,6}^{t}+\rho_{56}\left( {LC\_LC}_{r,5}^{t} \right)+\phi b({LC\_ND}_{r,6}^{t})+\phi c({D\_D}_{r,6}^{t})-\left( \varphi+\alpha+\mu+\rho_{67}+\rho_{68}+\rho_{69} \right){LC\_LC}_{r,6}^{t}$

$\boldsymbol{LC\_LC}_{\mathbf{r,7}}^{\mathbf{t+1}}={LC\_LC}_{r,7}^{t}+\rho_{57}\left( {LC\_LC}_{r,5}^{t} \right)+\rho_{67}\left( {LC\_LC}_{r,6}^{t} \right)+\phi b({LC\_ND}_{r,7}^{t})+\phi c({D\_D}_{r,7}^{t})-\left( \varphi+\alpha+\mu+\rho_{78}+\rho_{79} \right){LC\_LC}_{r,7}^{t}$

$\boldsymbol{LC\_LC}_{\mathbf{r,8}}^{\mathbf{t+1}}={LC\_LC}_{r,8}^{t}+\rho_{68}\left( {LC\_LC}_{r,6}^{t} \right)+\rho_{78}\left( {LC\_LC}_{r,7}^{t} \right)+\phi b({LC\_ND}_{r,8}^{t})+\phi c({D\_D}_{r,8}^{t})-\left( \varphi+\alpha+\mu\right){LC\_LC}_{r,8}^{t}$

$\boldsymbol{LC\_LC}_{\mathbf{r,9}}^{\mathbf{t+1}}={LC\_LC}_{r,9}^{t}+\rho_{69}\left( {LC\_LC}_{r,6}^{t} \right)+\rho_{79}\left( {LC\_LC}_{r,7}^{t} \right)$

**HCV treatment compartments**

$\boldsymbol{LC\_TR}_{\mathbf{r,0}}^{\mathbf{t+1}}={LC\_TR}_{r,0}^{t}+\alpha\left( {LC\_LC}_{r,0}^{t} \right)-\left( \mu+\frac{1}{\mathrm{Tt}_{\mathrm{acute}}} \right){LC\_TR}_{r,0}^{t}$

$for h\in\left[ 1,2,3,4,5,6,7,8 \right], \boldsymbol{LC\_TR}_{\mathbf{r,h}}^{\mathbf{t+1}}={LC\_TR}_{r,h}^{t}+\alpha\left( {LC\_LC}_{r,h}^{t} \right)-\left( \mu+\frac{1}{\mathrm{Tt}_{\mathrm{chronic}}} \right){LC\_TR}_{r,h}^{t}$

**HCV post treatment failure compartments**

$\boldsymbol{LC\_FT}_{\mathbf{r,0}}^{\mathbf{t+1}}={LC\_FT}_{r,0}^{t}+(1-pSVR)(\frac{1}{\mathrm{Tt}_{\mathrm{acute}}}){LC\_TR}_{r,0}^{t}-\left( \mu+\frac{1}{\mathrm{Ta}} \right){LC\_FT}_{r,0}^{t}$

$\boldsymbol{LC\_FT}_{\mathbf{r,1}}^{\mathbf{t+1}}={LC\_FT}_{r,1}^{t}+\left( 1-psc \right)\left( \frac{1}{\mathrm{Ta}} \right){LC\_FT}_{r,0}^{t}+(1-pSVR)(\frac{1}{\mathrm{Tt}_{\mathrm{chronic}}}){LC\_TR}_{r,1}^{t}-\left( \mu+\rho_{12} \right){LC\_FT}_{r,1}^{t}$

$for h\in\left[ 2,3,4 \right], \boldsymbol{LC\_FT}_{\mathbf{r,h}}^{\mathbf{t+1}}={LC\_FT}_{r,h}^{t}+\rho_{\left( h-1 \right)h}\left( {LC\_FT}_{r,h-1}^{t} \right)+(1-pSVR)(\frac{1}{\mathrm{Tt}_{\mathrm{chronic}}}){LC\_TR}_{r,h}^{t}-\left( \mu+\rho_{h\left( h+1 \right)} \right){LC\_FT}_{r,h}^{t}$

$\boldsymbol{LC\_FT}_{\mathbf{r,5}}^{\mathbf{t+1}}={LC\_FT}_{r,5}^{t}+\rho_{45}\left( {LC\_FT}_{r,4}^{t} \right)+(1-pSVR)(\frac{1}{\mathrm{Tt}_{\mathrm{chronic}}}){LC\_TR}_{r,5}^{t}-\left( \mu+\rho_{56}+\rho_{57} \right){LC\_FT}_{r,5}^{t}$

$\boldsymbol{LC\_FT}_{\mathbf{r,6}}^{\mathbf{t+1}}={LC\_FT}_{r,6}^{t}+\rho_{56}\left( {LC\_FT}_{r,5}^{t} \right)+(1-pSVR)(\frac{1}{\mathrm{Tt}_{\mathrm{chronic}}}){LC\_TR}_{r,6}^{t}-\left( \mu+\rho_{67}+\rho_{68}+\rho_{69} \right){LC\_FT}_{r,6}^{t}$

$\boldsymbol{LC\_FT}_{\mathbf{r,7}}^{\mathbf{t+1}}={LC\_FT}_{r,7}^{t}+\rho_{57}\left( {LC\_FT}_{r,5}^{t} \right)+\rho_{67}\left( {LC\_FT}_{r,6}^{t} \right)+(1-pSVR)(\frac{1}{\mathrm{Tt}_{\mathrm{chronic}}}){LC\_TR}_{r,7}^{t}-\left( \mu+\rho_{78}+\rho_{79} \right){LC\_FT}_{r,7}^{t}$

$\boldsymbol{LC\_FT}_{\mathbf{r,8}}^{\mathbf{t+1}}={LC\_FT}_{r,8}^{t}+\rho_{68}\left( {LC\_FT}_{r,6}^{t} \right)+\rho_{78}\left( {LC\_FT}_{r,7}^{t} \right)+(1-pSVR)(\frac{1}{\mathrm{Tt}_{\mathrm{chronic}}}){LC\_TR}_{r,8}^{t}-\left( \mu\right){LC\_FT}_{r,8}^{t}$

$\boldsymbol{LC\_FT}_{\mathbf{r,9}}^{\mathbf{t+1}}={LC\_FT}_{r,9}^{t}+\rho_{69}\left( {LC\_FT}_{r,6}^{t} \right)+\rho_{79}\left( {LC\_FT}_{r,7}^{t} \right)$

**HCV post-SVR compartments**

$\boldsymbol{LC\_ST}_{\mathbf{r,5}}^{\mathbf{t+1}}={LC\_ST}_{r,5}^{t}+pSVR(\frac{1}{\mathrm{Tt}_{\mathrm{chronic}}}){LC\_TR}_{r,5}^{t}-\left( \mu+\varepsilon_{56}+\varepsilon_{57} \right){LC\_ST}_{r,5}^{t}$

$\boldsymbol{LC\_ST}_{\mathbf{r,6}}^{\mathbf{t+1}}={LC\_ST}_{r,6}^{t}+pSVR(\frac{1}{\mathrm{Tt}_{\mathrm{chronic}}}){LC\_TR}_{r,6}^{t}+\varepsilon_{56}\left( {LC\_ST}_{r,5}^{t} \right)-\left( \mu+\varepsilon_{67}+\varepsilon_{68}+\varepsilon_{69} \right){LC\_ST}_{r,6}^{t}$

$\boldsymbol{LC\_ST}_{\mathbf{r,7}}^{\mathbf{t+1}}={LC\_ST}_{r,7}^{t}+pSVR(\frac{1}{\mathrm{Tt}_{\mathrm{chronic}}}){LC\_TR}_{r,7}^{t}+\varepsilon_{57}\left( {LC\_ST}_{r,5}^{t} \right)+\varepsilon_{67}\left( {LC\_ST}_{r,6}^{t} \right)-\left( \mu+\varepsilon_{78}+\varepsilon_{79} \right){LC\_ST}_{r,7}^{t}$

$\boldsymbol{LC\_ST}_{\mathbf{r,8}}^{\mathbf{t+1}}={LC\_ST}_{r,8}^{t}+pSVR(\frac{1}{\mathrm{Tt}_{\mathrm{chronic}}}){LC\_TR}_{r,8}^{t}+\varepsilon_{68}\left( {LC\_ST}_{r,6}^{t} \right)+\varepsilon_{78}\left( {LC\_ST}_{r,7}^{t} \right)-\left( \mu\right){LC\_ST}_{r,8}^{t}$

$\boldsymbol{LC\_ST}_{\mathbf{r,9}}^{\mathbf{t+1}}={LC\_ST}_{r,9}^{t}+\varepsilon_{69}\left( {LC\_ST}_{r,6}^{t} \right)+\varepsilon_{79}\left( {LC\_ST}_{r,7}^{t} \right)$

# **S3. Parameters estimations and model calibration**

**Table S1** summarizes the values for the parameters used in the model and the assumptions made. The details for initial distribution of the population and input parameters are described in Castry et al. [2].

Susceptible individuals could become infected with a force of infection $\lambda_{r}$. We assumed homogeneous mixing, i.e. no mixing preferences. The force of infection is therefore represented by the following equation: $\lambda_{r}=\beta_{r}\times I/N$, where $\beta_{r}$ represents the transmission rate for the risk group $r$, $I$ the number of infectious individuals and $N$ the total number of individuals in the population. We assumed that the force of infection was the same for HIV undiagnosed, HIV diagnosed or HIV linked to care MSM, as well as for primary and reinfections.

We assigned a relative risk of HCV transmission for the high-risk group ($\mathrm{RR}_{h}$) compared with the low-risk group. Thus, if $\beta_{l}$ ($\beta_{h}$) denotes the transmission rate for the low-risk (high-risk) group then $\beta_{h}=\beta_{l}\times{(RR}_{h})$. Thus, we only needed to estimate the transmission parameter for the low-risk group ($\beta_{l}$). For this purpose, we used Approximate Bayesian Computation (ABC), a Bayesian method used to infer some parameters of a model without likelihood estimation [3]. We used the package “abc” [4] of the statistical software R.

Briefly, the main idea of ABC is to repeatedly sample a parameter value, $\theta_{i}$, from its prior distribution to simulate a dataset, $y_{i}$, for $i$ = 1,…,$n$ where $n$ is the number of simulations. Then, we compute the value of a summary statistic $s_{i}$ and compare it to the value of the summary statistic obtained from the actual data, $s_{\mathrm{obs}}$, using a distance measure $d$. If the distance between $s_{\mathrm{obs}}$ and $s_{i}$ is less than $d$, the parameter value $\theta_{i}$ is accepted. A threshold for $d$ is usually obtained by providing the tolerance rate $\omega$, defined as the proportion of accepted simulations. In our case, the parameter of interest was $\beta_{l}$ (transmission parameter for the low-risk group) and the informative summary statistic used to infer its value was the primary HCV incidence between 2014 and 2017 [5]. We performed 500,000 simulations and the tolerance threshold $\omega$ was set at 0.1. The prior law distribution was $\beta_{l}\sim U[0 ;0.02]$ where $U$ is a uniform law on [0; 0.02]. We found the following results: $\beta_{l}$ = 0.0120 [95% CI: 0.0111 – 0.0129].

# **S4. Cost data**

HCV cost inputs were limited to direct medical costs associated with HCV screening, HCV care and HCV treatment.

Costs of HCV screening included the test for HCV antibodies (€12.96), and when positive, a HCV-RNA screening test (€54) to confirm active infection. Individuals susceptible to reinfection after treatment success continued to undergo HCV surveillance with PCR tests but not with additional serological tests. We also included diagnosis costs, i.e. initial checkup at the time of HCV diagnosis.

To estimate resource consumption for HCV care, ambulatory and hospital costs stratified by disease stage were obtained from a study on healthcare consumption for chronic hepatitis C in France (**Table S3**) [9].

Costs of HCV treatment included costs associated with antiviral treatment. Following the French treatment recommendations and recent drug prices, we considered a single price of €24,935 for a 12-week DAA cure, with the exception of patients with decompensated cirrhosis who also receive 12 weeks of ribavirin leading to a total cost of €25,750 [10].

The yearly costs of HIV care (€17,529), which include antiretroviral treatment, were obtained from two previous economic studies conducted in France [11,12].

Regarding the risk reduction intervention, we used the hourly wage for hospital nurses at Bichat Hospital in Paris (€28, data not published) to estimate the costs related to counseling sessions. Note that initial startup costs and overhead expenses – including program promotion, patient recruitment, staff training, and facilities – were not included in the analysis. However, costs of patient recruitment are probably minimal fur such interventions because the referring clinicians during HIV care are supposed to refer patients to counseling sessions.

# **S5. Sensitivity analysis assuming assortative mixing**

We performed a sensitivity analysis, assuming that most sexual partnerships occurred within the same risk group (i.e. assortative mixing). Here, the force of infection depends on the mixing matrix:

$$\sigma=\left( \begin{matrix} \sigma_{\mathrm{hh}} & \sigma_{\mathrm{hl}} \\ \sigma_{\mathrm{lh}} & \sigma_{\mathrm{ll}} \end{matrix} \right)$$

Where$\sigma_{rr^{'}}$ depicts the probability for an $\boldsymbol{r}$-risk individual to have a sexual partnership with an $r^{'}$-risk individual (with $r \epsilon\{l,h\}$ corresponding to low-risk and high-risk group). The elements of the mixing matrix verify $0<\sigma_{rr^{'}}<1$ and$\sum_{r} \sigma_{rr^{'}}=1$

In this theoretical analysis, we assumed that 90% of partnerships were formed between individuals within the same risk group, that is:

$$\sigma=\left( \begin{matrix} 0.9 & 0.1 \\ 0.1 & 0.9 \end{matrix} \right)$$

Because we lacked data on the contact rate (or total number of partnerships) of MSM according to their risk behavior, we used a simplified form for the force of infection:

$$\lambda_{r}=\sum_{r^{'}\epsilon\{h,l\}} \frac{\sigma_{rr^{'}}\beta_{r^{'}}I_{r^{'}}}{N_{r^{'}}}$$

where, for each risk group $r^{'}$, $I_{r^{'}}$ was the number of infectious individuals, $N_{r^{'}}$ was the total population, and $\beta_{r^{'}}$ represented the transmission parameter ~~from~~ for risk group $r^{'}$.

As for the main analysis, we assigned a relative risk of HCV transmission from the high-risk group ($\mathrm{RR}_{h}$) compared with the low-risk group so that $\beta_{h}=\beta_{l}\times{(RR}_{h})$. The transmission parameter from the low-risk group ($\beta_{l}$) was determined via calibration to reflect primary HCV incidence observed between 2014 and 2017 among MSM living with HIV in care.

# **S6. Supplementary figures and tables**

**Figure S1. Flow diagrams of the HCV transmission and progression model.**

**Notes:** (A) HCV infection and HCV and HIV cascades of care. Each compartment was stratified into two risk groups (subscript $r$). White compartments were HCV susceptible individuals (i.e. HCV-uninfected). Gray compartments represented HCV-infected MSM; these compartments were also stratified according to hepatitis C disease progression. (subscript $h$) (B) Hepatitis C disease progression model. After acute infection, METAVIR scores were used to classify stages of liver disease: F0 = no fibrosis; F1 = portal fibrosis without septa; F2 = portal fibrosis with septa; F3 = numerous septa without cirrhosis; F4 = cirrhosis. The second part of the model described cirrhosis complications: decompensated cirrhosis, hepatocellular carcinoma, liver transplantation. Those complications led to death related to HCV infection. **Abbreviations**: HIV+, living with HIV; HCV-, HCV uninfected; HCV+, HCV-infected; ND_X, HIV undiagnosed and HCV uninfected; D_X, HIV-diagnosed and HCV uninfected; LC_X, linked to HIV care and HCV uninfected; ND_ND, undiagnosed for both infections; D_D, diagnosed for both infections; D_ND, HIV-diagnosed and HCV undiagnosed; LC_ND, linked to HIV care and HCV undiagnosed; LC_LC, linked to care for both infections; LC_TR, linked to HIV care and under HCV treatment; LC_FT, linked to HIV care and HCV post treatment failure; LC_ST, linked to HIV care and post SVR (for stages F4, decompensated cirrhosis and hepatocellular carcinoma).

**Table S1. Model parameters values**

| Parameter | Value | Sensitivity analysis | References/Details |
| --- | --- | --- | --- |
| Initial population size (2014) | 53,200 |  | [15] |
| Initial distribution across the HIV cascade of care | | | |
| MSM living with HIV, undiagnosed | 9,200 (17%) | 7,800 (15%) -  11,200 (21%) | [15] Range for sensitivity analysis based on 95% CI on number undiagnosed and diagnosed non-linked to care. Number in HIV care was fixed in order to keep total population of 53,200 |
| MSM living with HIV, diagnosed (not in care) | 2,700 (5%) | 2,200 (4%) -  3,300 (6%) |  |
| MSM living with HIV, in care | 41,300 (78%) | 43,200 (81%) - 38,700 (73%) |  |
| Initial prevalence of chronic active infection (HCV RNA+) | | | |
| Overall prevalence among MSM living with HIV | 3.62% | 3.26% - 3.98% | Data from the ANRS CO4-FHDH cohort and the PREVAGAY 2015 study; additional use of published data regarding the proportion of HCV RNA positive among anti-HCV positive [16] Range for sensitivity analysis: assumption (+/- 10% of the base case value) |
| Prevalence among MSM living with HIV, in care | 4.00% | 3.60% - 4.40% | Among this population (in HIV-care), we estimated the prevalence at 0.55% in the low-risk group and 12.86% in the high-risk group. Range for sensitivity analysis: assumption (+/- 10% of the base case value) |
| Prevalence among MSM living with HIV, not in care | 2.31% | 2.08% - 2.54% | Among this population (not in HIV-care), we estimated the prevalence at 0.32% in the low-risk group and 7.42% in the high-risk group. Values for sensitivity analysis: assumption (+/-10% of the base case value) |
| Initial distribution across the HCV cascade of care (2014) | | | |
| Distribution among HIV-diagnosed, non-linked to care MSM, between HCV undiagnosed (D_ND) and HCV-diagnosed (D_D) | 50% | 25% - 75% | Assumption (% for D_ND) |
| Distribution among MSM in HIV-care, between HCV undiagnosed (LC_ND) and HCV-care (LC_LC) | 50% | 25% - 75% | Assumption (% for LC_ND) |
| Initial distribution in the natural history model (2014) | | | |
| Acute hepatitis C | 0% |  | Assumption |
| F0 | 30% | 20% - 40% |  |
| F1 | 25% | 20% - 30% |  |
| F2 | 25% | 20% - 25% |  |
| F3 | 10% | 20% - 5% |  |
| F4 | 10% | 20% - 0% |  |
| Decompensated cirrhosis | 0% |  |  |
| Hepatocellular carcinoma | 0% |  |  |
| Liver transplantation | 0% |  |  |
| Parameters for HCV transmission | | | |
| Monthly HCV transmission rate for the low-risk group ($\boldsymbol{\beta}_{\mathbf{l}}$) | 0.0120 | 0.0111 - 0.0129 | Model calibrated to primary HCV incidence among MSM living with HIV over 2014-2017, from the ANRS CO4-FHDH cohort. Range for sensitivity analysis based on the 95% CI from the estimated parameter |
| Proportion of individuals in the high-risk group (chemsex practices) | 28% | 22% - 36% | PREVAGAY 2015. Range for sensitivity analysis based on the 95% CI from the estimated parameter |
| Relative risk of HCV transmission for the high-risk group compared with the low-risk group | 5.60 | 3.90 - 8.00 | PREVAGAY 2015. Range for sensitivity analysis: derived from the 95% CI of both rates (ratios between lower and upper bounds) |
| Entry | | | |
| Yearly number of new HIV infections among MSM ($\boldsymbol{\pi}$) (model entry) | 2014: 2,726  2015: 2,634  2016: 2,577  2017: 2,587  2018: 2,501 |  | 2014-2018 estimates were obtained from surveillance data on newly diagnosed HIV cases and back-calculation modelling.  After 2018, linear extrapolation of the 2014-2018 decreasing trend |
| Proportion of HCV coinfected among newly HIV-infected MSM ($\boldsymbol{\pi'}$) | 2% | 1% - 3% | Based on surveillance data from Santé publique France [17]. Range for sensitivity analysis: assumption |
| Background mortality | | | |
| General mortality rate |  |  | Depends on age the mean age in the population, starting at 40 years old in 2014, then increasing by one every year. Mortality rates based on Human Mortality Database [18] |
| Excess mortality rate due to HIV infection | 0.0061 y^-1^ | 0.0048 - 0.0074 y^-1^ | Excess mortality rate due to HIV infection in the CASCADE cohort, period 2006-2008 [19]. Range for sensitivity analysis based on CI from the study |
| Parameters for HIV and HCV cascade of care | | | |
| Time from HIV infection to HIV diagnosis ($\boldsymbol{\tau a}$) | 2.7 years | 2 years - 3.5 years | [15,20] Estimated from surveillance data on newly diagnosed HIV cases and back-calculation modelling. Range for sensitivity analysis: assumption. Same time interval was used for the time from HIV-HCV coinfection to HIV and HCV diagnosis ($\tau b$) |
| Time from HIV diagnosis to entry into HIV care ($\boldsymbol{\phi a}$) | 10 days | 3 days - 22 days | Supervie (personal communication). Estimated from the FHDH hospital-based cohort for 2014-2016. Range for sensitivity analysis based on IQR from the estimated time. Same time interval was used for the time from HIV and HCV diagnosis to entry into care for both infections ($\phi c$) |
| Time from HCV infection to HCV diagnosis, for MSM in HIV care ($\boldsymbol{\phi b}$) | 1 year |  | Based on the estimate of HCV screening frequency among MSM living with HIV enrolled in the FHDH hospital-based cohort [5]. Value was changed for HCV screening strategies |
| Time from entry into HCV care to HCV treatment ($\boldsymbol{\alpha}$) | 1 month | 1 month - 3 months | Rapid treatment initiation (from 2019) [21] |
| Loss to follow-up rate ($\boldsymbol{\varphi}$) | 0.015 y^-1^ | 0.010 y^-1^ - 0.020 y^-1^ | Assumption (expert opinion) |
| Parameters for HCV treatment | | | |
| Duration of treatment, acute infection | 8 weeks |  | [22] |
| Duration of treatment, chronic infection | 12 weeks |  | [22] |
| Efficacy of HCV treatment with DAAs (Psvr) | 95% | +/- 5% | Rounded from [23] (93.5% observed among patients living with HIV, 93.8% among those without cirrhosis). Depends on the period and the disease stage (**Table S2**). Range: assumption |
| Parameters for HCV disease progression | | | |
| Duration of acute hepatitis C infection until spontaneous clearance (Ta) | 6 months |  | [24] |
| Probability of spontaneous clearance during acute infection (psc) | 15% | 15% - 25% | [24] Range for sensitivity analysis: assumption (could be higher than 15%, close to the rate observed among HCV mono-infected population) |
| Transition probability F0 ⇾ F1 ($\boldsymbol{\rho}_{\mathbf{12}}$) | 0.122 y^-1^ | 0.098 y^-1^ - 0.153 y^-1^ | [25]. Range for sensitivity analysis based on CI from the study |
| Transition probability F1 ⇾ F2 ($\boldsymbol{\rho}_{\mathbf{23}}$) | 0.115 y^-1^ | 0.095 y^-1^ - 0.140 y^-1^ |  |
| Transition probability F2 ⇾ F3 ($\boldsymbol{\rho}_{\mathbf{34}}$) | 0.124 y^-1^ | 0.097 y^-1^ - 0.159 y^-1^ |  |
| Transition probability F3 ⇾ F4 ($\boldsymbol{\rho}_{\mathbf{45}}$) | 0.115 y^-1^ | 0.098 y^-1^ - 0.135 y^-1^ |  |
| Transition rate F4 ⇾ DC ($\boldsymbol{\rho}_{\mathbf{56}}$) | 0.0339 y^-1^ | 0.0288 y^-1^ - 0.0390 y^-1^ | Inferred from [26]: liver decompensation 5-year cumulative incidence (from compensated cirrhosis = 15.6%). Range for sensitivity analysis: assumption (+/- 15% of the base case value) |
| Transition rate F4 ⇾ HCC ($\boldsymbol{\rho}_{\mathbf{57}}$) | 0.0283 y^-1^ | 0.0240 y^-1^ - 0.0325 y^-1^ | Inferred from [26]: HCC 5-year cumulative incidence (from compensated cirrhosis = 13.2%). Range for sensitivity analysis: assumption (+/- 15% of the base case value) |
| Transition rate DC ⇾ HCC ($\boldsymbol{\rho}_{\mathbf{67}}$) | 0.0283 y^-1^ | 0.0240 y^-1^ - 0.0325 y^-1^ | Assumption: same as transition from F4 to HCC |
| Transition probability DC ⇾ LT ($\boldsymbol{\rho}_{\mathbf{68}}$) | 0.120 | 0.096 - 0.144 | [27,28]. Range for sensitivity analysis: assumption (+/- 20% of the base case value) |
| Transition probability HCC ⇾ LT ($\boldsymbol{\rho}_{\mathbf{78}}$) | 0.170 | 0.136 - 0.204 |  |
| Transition rate DC ⇾ HCV-related death ($\boldsymbol{\rho}_{\mathbf{69}}$) | 0.306 y^-1^ | 0.260 y^-1^ - 0.352 y^-1^ | [29,30]. Ranges for sensitivity analysis: assumption (+/- 15% of the base case value) |
| Transition rate HCC ⇾ HCV-related death ($\boldsymbol{\rho}_{\mathbf{79}}$) | 0.433 y^-1^ | 0.368 y^-1^ - 0.498 y^-1^ |  |
| Transition probability LT ⇾ HCV-related death ($\boldsymbol{\rho}_{\mathbf{89}}$) | 0.032 | 0.026 - 0.038 | [31]. Range for sensitivity analysis: assumption (+/- 20% of the base case value) |
| Decreased progression to DC hazard ratio for HCV infection (with cirrhosis) due to SVR | 0.51 | 0.51 - 1 | Hazard ratios from [32]. Ranges for sensitivity analysis: assumption (no impact of SVR on progression) |
| Decreased progression to HCC hazard ratio for HCV infection due to SVR | 0.44 | 0.44 - 1 |  |
| Decreased liver-related mortality hazard ratio for HCV infection due to SVR | 0.21 | 0.21 - 1 |  |

**Abbreviations**: CI, Confidence interval; DC, Decompensated cirrhosis; HCC, Hepatocellular carcinoma; LT, Liver transplant; SVR, Sustained virological response; y^-1^, per year.

**Table S2. Assumptions for HCV treatment uptake evolution among MSM living with HIV in France from 2014 to 2019**

| Period | Disease state | Eligibility to treatment | SVR rate (pSVR) | Time from entry into HCV care to treatment initiation ($\boldsymbol{\alpha}$) |
| --- | --- | --- | --- | --- |
| 2014-2015 | Acute infection | Yes | 85% | 3 months |
|  | F0-F2 | Yes | 95% | 1 year |
|  | F3 | Yes | 95% | 6 months |
|  | F4 | Yes | 90% | 6 months |
|  | DC | No | - | **-** |
|  | HCC | No | - | **-** |
| 2016-2017 | Acute infection | Yes | 95% | 3 months |
|  | F0-F2 | Yes | 95% | 6 months |
|  | F3 | Yes | 95% | 6 months |
|  | F4 | Yes | 95% | 3 months |
|  | DC | Yes | 85% | 3 months |
|  | HCC | No | **-** | **-** |
| 2018 | Acute infection | Yes | 95% | 1 month |
|  | F0-F2 | Yes | 95% | 3 months |
|  | F3 | Yes | 95% | 3 months |
|  | F4 | Yes | 95% | 3 months |
|  | DC | Yes | 85% | 3 months |
|  | HCC | No | - | - |
| 2019 | Acute infection | Yes | 95% | 1 month |
|  | F0-F2 | Yes | 95% | 1 month |
|  | F3 | Yes | 95% | 1 month |
|  | F4 | Yes | 95% | 1 month |
|  | DC | Yes | 85% | 1 month |
|  | HCC | No | - | - |

**Notes**: Values from 2019 were kept unchanged thereafter. **Abbreviations**: DC, Decompensated cirrhosis; HCC, Hepatocellular carcinoma; SVR, Sustained viral response.

**Table S3. Annual mean costs attributable to chronic hepatitis C: ambulatory costs (never treated and after HCV treatment failure) and hospitalization costs (no death and in-hospital death) [9]**

| Liver disease stage | Ambulatory costs, mean € | | Hospitalization costs, mean € | |
| --- | --- | --- | --- | --- |
|  | **Never-treated** | **After treatment failure** | **No death** | **In-hospital death** |
| Fibrosis F0 to F2 | 78 | 59 | 367 | 586 |
| Fibrosis F3 | 142 | 96 | 367 | 586 |
| Compensated cirrhosis (F4) | 253 | 79 | 1,853 | 10,055 |
| Decompensated cirrhosis | | | | |
| First year | 107 | 107 | 10,702 | 14,455 |
| Following years | 107 | 107 | 18,092 | 21,576 |
| HCC | 107 | 107 | 15,073 | 21,524 |
| Liver transplant | | | | |
| First year | 0 | 0 | 63,642 | 92,806 |
| Following years | 0 | 0 | 7,009 | 16,480 |

**Notes**: Costs are expressed in 2020 euros. HCC, Hepatocellular carcinoma.

**Table S4. Health-related utilities**

|  | Value | References |
| --- | --- | --- |
| Health-related utilities HIV mono-infected individuals | 0.94 | [33] |
| Health-related utilities in HIV-infected chronic hepatitis C patients* | | |
| Fibrosis F0 to F1 | 0.77 | [33,34] |
| Fibrosis F2 | 0.76 | [33,34] |
| Fibrosis F3 to F4 | 0.70 | [33,34] |
| Decompensated cirrhosis / Hepatocellular carcinoma | 0.54 | [14,33,34] |
| Liver transplant | 0.76 | [14,33,34] |
| Health-related utilities in patients after sustained virological response* | | |
| Sustained virological response from F0-F3 | 0.94 | Assumption: health utility score returned to that of an HIV mono-infected |
| Sustained virological response from F4 | 0.79 | [14,33] |

**Notes**: Quality of life value in HIV-infected chronic hepatitis C patients was obtained by subtracting 0.06 points to the estimated value for HCV mono-infected patients (for example, the health-related utility in fibrosis stage F2 corresponded to 0.82-0.06=0.76). Health utilities were left unchanged during DAA treatment. After clearing the HCV infection up to fibrosis stage F3, we assumed that the health utility score returned to that of an HIV mono-infected person (i.e. 0.94). Furthermore, health utility was increased after SVR for F4, as observed in Adelphi study [14].

**Table S5. Distributions used in the probabilistic sensitivity analysis**

| Input parameter | Point estimate | Probability distribution | Distribution parameters | Source |
| --- | --- | --- | --- | --- |
| Relative risk of HCV transmission for the high-risk group | 5.60 | Log-normal | Mean = 1.72  SD = 0.18 | PREVAGAY 2015 |
| Proportion of  high-risk MSM | 0.28 | Beta | $\text{α}$ = 521  $\text{β}$ = 1,318 | PREVAGAY 2015 (estimated from proportions) |
| Proportion of HCV coinfected among newly HIV-infected MSM | 0.02 | Beta | $\text{α}$ = 15.04  $\text{β}$ = 736.91 | Assumption, to obtain 95% CI [0.01 - 0.02] (fitting beta distribution by method of moments) |
| HCV ambulatory costs, fibrosis F0 to F2,  never-treated | €78 | Gamma | $\text{α}$ = 50.28  $\text{β}$ = 1.55 | [9] Based on mean (€78) and SD (11), fitting gamma distribution by method of moments |
| HCV ambulatory costs, fibrosis F0 to F2,  after treatment failure | €59 | Gamma | $\text{α}$ = 20.60  $\text{β}$ = 2.86 | [9] Based on mean (€59) and SD (13), fitting gamma distribution by method of moments |
| HCV ambulatory costs, fibrosis F3, never-treated | €142 | Gamma | $\text{α}$ = 35.00  $\text{β}$ = 4.06 | [9] Based on mean (€142) and SD (24), fitting gamma distribution by method of moments |
| HCV ambulatory costs, fibrosis F3,  after treatment failure | €96 | Gamma | $\text{α}$ = 31.89  $\text{β}$ = 3.01 | [9] Based on mean (€96) and SD (17), fitting gamma distribution by method of moments |
| HCV ambulatory costs, fibrosis F4, never-treated | €253 | Gamma | $\text{α}$ = 132.89  $\text{β}$ = 1.91 | [9] Based on mean (€253) and SD (22), fitting gamma distribution by method of moments |
| HCV ambulatory costs, fibrosis F4,  after treatment failure | €79 | Gamma | $\text{α}$ = 15.60  $\text{β}$ = 5.06 | [9] Based on mean (€79) and SD (20), fitting gamma distribution by method of moments |
| HCV ambulatory costs, DC-HCC, never treated or after treatment failure | €107 | Gamma | $\text{α}$ = 21.64  $\text{β}$ = 4.94 | [9] Based on mean (€107) and SD (23), fitting gamma distribution by method of moments |
| HCV hospitalization costs, fibrosis F0 to F3, no-death | €367 | Triangular | Mode = 367  Min = 257  Max = 477 | [9] for point estimate. Assumption for triangular distribution so that mode corresponds to point estimate, and max/min correspond to +/- 30% of point estimate |
| HCV hospitalization costs, fibrosis F0 to F3, extra cost due to death | €219 | Triangular | Mode = 219  Min = 153  Max = 285 | [9] for point estimate. Assumption for triangular distribution so that the mode corresponds to point estimate, and max/min correspond to +/- 30% of point estimate |
| HCV hospitalization costs, fibrosis F4,  no-death | €1,853 | Log-normal | $\text{μ}$ = 6.37  $\text{σ}$ = 1.52 | [9]. Estimated from mean (€1,853) and median (€586) |
| HCV hospitalization costs, fibrosis F4,  in-hospital death | €8,202 | Log-normal | $\text{μ}$ = 8.58  $\text{σ}$ = 0.93 | [9]. Estimated from mean (€8,202) and median (€5,322) |
| HCV hospitalization costs, DC, 1^st^ year, no-death | €10,702 | Log-normal | $\text{μ}$ = 8.86  $\text{σ}$ = 0.91 | [9]. Estimated from mean (€10,702) and median (€7,052) |
| HCV hospitalization costs, DC, 1^st^ year,  extra cost due to death | €3,753 | Log-normal | $\text{μ}$ = 8.14  $\text{σ}$ = 0.41 | [9]. Estimated from mean (€3,753) and median (€3,443) |
| HCV hospitalization costs, DC, following years, no-death | €18,092 | Log-normal | $\text{μ}$ = 9.45  $\text{σ}$ = 0.84 | [9]. Estimated from mean (€18,092) and median (€12,675) |
| HCV hospitalization costs, DC, following years, extra cost due to death | €3,484 | Log-normal | $\text{μ}$ = 8.08  $\text{σ}$ = 0.38 | [9]. Estimated from mean (€3,484) and median (€3,242) |
| HCV hospitalization costs, HCC, no-death | €15,073 | Log-normal | $\text{μ}$ = 9.35  $\text{σ}$ = 0.74 | [9]. Estimated from mean (€15,073) and median (€11,464) |
| HCV hospitalization costs, HCC, extra cost due to death | €6,451 | Log-normal | $\text{μ}$ = 8.67  $\text{σ}$ = 0.45 | [9]. Estimated from mean (€6,451) and median (€5,826) |
| HCV hospitalization costs, liver transplant, 1^st^ year, no-death | €63,642 | Log-normal | $\text{μ}$ = 10.87  $\text{σ}$ = 0.61 | [9]. Estimated from mean (€63,642) and median (€52,830) |
| HCV hospitalization costs, liver transplant, 1^st^ year, extra cost due to death | €31,638 | Log-normal | $\text{μ}$ = 10.28  $\text{σ}$ = 0.40 | [9]. Estimated from mean (€31,638) and median (€29,164) |
| HCV hospitalization costs, liver transplant, following years, no-death | €7,009 | Log-normal | $\text{μ}$ = 7.87  $\text{σ}$ = 1.41 | [9]. Estimated from mean (€7,009) and median (€2,607) |
| HCV hospitalization costs, liver transplant, following years, extra cost due to death | €9,471 | Log-normal | $\text{μ}$ = 8.78  $\text{σ}$ = 0.86 | [9]. Estimated from mean (€9,471) and median (€6,534) |
| HIV care costs | €17,529 | Triangular | Mode = 17,529  Min = 12,270  Max = 22,788 | Assumption for triangular distribution so that the mode corresponds to point estimate, and max/min correspond to +/- 30% of point estimate |
| Risk reduction intervention cost (hourly wage for hospital nurses) | €28 | Triangular | Mode = 28  Min = 20  Max = 36 | Assumption for triangular distribution so that the mode corresponds to point estimate, and max/min correspond to +/- 30% of point estimate |
| Cost of HCV serology test | €12.96 | Triangular | Mode = 12.96  Min = 9.07  Max = 16.85 | Assumption for triangular distribution so that the mode corresponds to point estimate, and max/min correspond to +/- 30% of point estimate |
| Cost of HCV PCR test | €54 | Triangular | Mode = 54  Min = 38  Max = 70 | Assumption for triangular distribution so that the mode corresponds to point estimate, and max/min correspond to +/- 30% of point estimate |
| HIV-related utility (used to estimate decrease in utility due to HIV infection, as 1 minus the point estimate) | 0.94 | Beta | $\text{α}$ = 415.73  $\text{β}$ = 26.54 | [33] for point estimate. Assumption for Beta distribution, to obtain 95% CI range of +/- 20% of the point estimate (fitting beta distribution by method of moments) |
| HCV-related utility, fibrosis F0 to F1 | 0.83 | Beta | $\text{α}$ = 146.23  $\text{β}$ = 29.95 | [34] for point estimate. Assumption for Beta distribution, to obtain 95% CI range of +/- 20% of the point estimate (fitting beta distribution by method of moments) |
| HCV-related utility, fibrosis F2 | 0.82 | Beta | $\text{α}$ = 138.07  $\text{β}$ = 30.31 | [34] for point estimate. Assumption for Beta distribution, to obtain 95% CI range of +/- 20% of the point estimate (fitting beta distribution by method of moments) |
| HCV-related utility, fibrosis F3 to F4 | 0.76 | Beta | $\text{α}$ = 103.41  $\text{β}$ = 32.65 | [34] for point estimate. Assumption for Beta distribution, to obtain 95% CI range of +/- 20% of the point estimate (fitting beta distribution by method of moments) |
| HCV-related utility, DC-HCC | 0.60 | Beta | $\text{α}$ = 61.90  $\text{β}$ = 41.27 | [14,34] for point estimate. Assumption for Beta distribution, to obtain 95% CI range of +/- 20% of the point estimate (fitting beta distribution by method of moments) |
| HCV-related utility, liver transplant | 0.82 | Beta | $\text{α}$ = 138.07  $\text{β}$ = 30.31 | [14,34] for point estimate. Assumption for Beta distribution, to obtain 95% CI range of +/- 20% of the point estimate (fitting beta distribution by method of moments) |
| HCV-related utility, sustained virological response from F4 | 0.85 | Beta | $\text{α}$ = 165.82  $\text{β}$ = 29.26 | [14] for point estimate. Assumption for Beta distribution, to obtain 95% CI range of +/- 20% of the point estimate (fitting beta distribution by method of moments) |

**Abbreviations**: DC, Decompensated cirrhosis; HCC, Hepatocellular carcinoma.

**Table S6. Description of costs, number of infections, and cost per infection averted for non-dominated strategies on the efficiency frontier**

| Strategy | Costs (€) | Number of infections | Incremental costs (compared to previous strategy) | Prevented infections (compared to previous strategy) | Cost per infection averted (€) |
| --- | --- | --- | --- | --- | --- |
| S5 = screening every year; risk reduction  (high-risk) | 32,884,734,729 | 2,583 | - | - | - |
| S7 = screening every 6 months; risk reduction (high-risk) | 23,899,775,000 | 2,185 | 15,040,271 | 398 | 37,790 |
| S8 = screening every 6 months; risk reduction (all) | 23,908,925,320 | 1,966 | 9,150,320 | 219 | 41,782 |
| S10 = screening every 3 months; risk reduction (all) | 23,950,032,800 | 1,882 | 41,107,480 | 84 | 489,375 |

**Notes**: The strategies presented correspond to the four non-dominated strategies (i.e. those on the efficiency frontier) in the cost per QALY analysis (cf. Table 3 and Figure 1 of the main manuscript). They were sorted by increasing costs. In contrast to Table 3 of the main manuscript and Tables S7, S8, S9 below, cumulative costs for the whole cohort throughout the simulation were presented instead of lifetime costs in order to ensure comparability with the number of infections and to calculate the costs per infection averted. Costs and number of infections were discounted.

**Table S7. Sensitivity analysis decreasing the efficacy of risk reduction strategies to 15% and 10%**

| Strategy | Lifetime costs  (€) | Life expectancy (LYs) | Quality-adjusted life expectancy (QALYs) | Number of infections (reinfections after SVR) | ICER (€/LY) | ICER (€/QALY) |
| --- | --- | --- | --- | --- | --- | --- |
| 15% risk reduction | | | | | | |
| S5 = screening every year; risk reduction (high-risk) | 351,546 | 20.2404 | 18.9914 | 2,752 (362) | - | - |
| S1 = screening every year | 351,646 | 20.2403 | 18.9893 | 3,320 (484) | SD | SD |
| S6 = screening every year; risk reduction (all) | 351,679 | 20.2405 | 18.9923 | 2,513 (340) | 1,330,000 | ED |
| S7 = screening every 6 months; risk reduction (high-risk) | 351,754 | 20.2405 | 18.9953 | 2,307 (295) | SD | 53,333 |
| S2 = screening every 6 months | 351,807 | 20.2404 | 18.9942 | 2,701 (378) | SD | SD |
| S8 = screening every 6 months; risk reduction (all) | 351,905 | 20.2406 | 18.9958 | 2,135 (282) | 2,260,000 | 302,000 |
| S9 = screening every 3 months (high-risk); screening every 6 months (low-risk); risk reduction (high-risk) | 351,965 | 20.2405 | 18.9959 | 2,233 (285) | SD | 600,000 |
| S3 = screening every 3 months (high-risk); screening every 6 months (low-risk) | 352,013 | 20.2405 | 18.9949 | 2,597 (362) | SD | SD |
| S4 = screening every 3 months | 352,406 | 20.2405 | 18.9953 | 2,550 (354) | SD | SD |
| S10 = screening every 3 months; risk reduction (all) | 352,513 | 20.2406 | 18.9967 | 2,038 (268) | SD | 685,000 |
| 10% risk reduction | | | | | | |
| S5 = screening every year; risk reduction (high-risk) | 351,603 | 20.2404 | 18.9908 | 2,931 (400) | - | - |
| S1 = screening every year | 351,646 | 20.2403 | 18.9893 | 3,320 (484) | SD | SD |
| S6 = screening every year; risk reduction (all) | 351,758 | 20.2404 | 18.9914 | 2,761 (383) | SD | ED |
| S7 = screening every 6 months; risk reduction (high-risk) | 351,797 | 20.2405 | 18.9949 | 2,434 (321) | 1,940,000 | 47,317 |
| S2 = screening every 6 months | 351,807 | 20.2404 | 18.9942 | 2,701 (378) | SD | SD |
| S8 = screening every 6 months; risk reduction (all) | 351,966 | 20.2405 | 18.9953 | 2,314 (312) | SD | ED |
| S9 = screening every 3 months (high-risk); screening every 6 months (low-risk); risk reduction (high-risk) | 352,007 | 20.2405 | 18.9956 | 2,351 (309) | SD | 300,000 |
| S3 = screening every 3 months (high-risk); screening every 6 months (low-risk) | 352,013 | 20.2405 | 18.9949 | 2,597 (362) | SD | SD |
| S4 = screening every 3 months | 352,406 | 20.2405 | 18.9953 | 2,550 (354) | SD | SD |
| S10 = screening every 3 months; risk reduction (all) | 352,571 | 20.2405 | 18.9962 | 2,201 (295) | SD | 940,000 |

**Notes**: The strategies were sorted by increasing costs. Incremental costs, incremental QALYs and ICER were all compared to the previous relevant strategy (i.e. not dominated). Note that lifetime costs, LYs, and QALYs are presented on average per person, while the number of infections (and reinfections after SVR) are cumulative for the whole cohort throughout the simulation between 2021 and 2065. SD, strongly dominated (more expensive and less or equally effective). SD, strongly dominated; ED, extendedly dominated; ICER, incremental cost-effectiveness ratio; QALY, quality-adjusted life year; SVR, sustained virological response. Strongly dominated = more expensive and less (or equally) effective. Extendedly dominated = at least one more expensive strategy has a lower ICER.

**Table S8. Sensitivity analysis assuming a 98% SVR rate from 2019 for those treated before cirrhosis (i.e., acute infection and fibrosis stages F0, F1, F2, F3)**

| Strategy | Lifetime costs  (€) | Life expectancy (LYs) | Quality-adjusted life expectancy (QALYs) | Number of infections  (reinfections after SVR) | ICER (€/LY) | ICER (€/QALY) |
| --- | --- | --- | --- | --- | --- | --- |
| S5 = screening every year; risk reduction (high-risk) | 351,323 | 20.2415 | 18.9986 | 2,064 (250) | - | - |
| S1 = screening every year (current practices) | 351,406 | 20.2415 | 18.9972 | 2,577 (351) | SD | SD |
| S6 = screening every year; risk reduction (all) | 351,464 | 20.2415 | 18.9992 | 1,849 (235) | SD | ED |
| S7 = screening every 6 months; risk reduction (high-risk) | 351,538 | 20.2416 | 19.0014 | 1,757 (210) | 2,150,000 | 77,338 |
| S2 = screening every 6 months (current guidelines) | 351,607 | 20.2415 | 19.0006 | 2,120 (281) | SD | SD |
| S8 = screening every 6 months; risk reduction (all) | 351,710 | 20.2416 | 19.0017 | 1,598 (201) | SD | 573,333 |
| S9 = screening every 3 months (high-risk); screening every 6 months (low-risk); risk reduction (high-risk) | 351,778 | 20.2416 | 19.0016 | 1,705 (203) | SD | SD |
| S3 = screening every 3 months (high-risk); screening every 6 months (low-risk) | 351,818 | 20.2415 | 19.0012 | 2,042 (269) | SD | SD |
| S4 = screening every 3 months | 352,211 | 20.2415 | 19.0014 | 2,006 (264) | SD | SD |
| S10 = screening every 3 months; risk reduction (all) | 352,331 | 20.2416 | 19.0022 | 1,531 (192) | SD | 1,242,000 |

**Notes**: The strategies were sorted by increasing costs. Incremental costs, incremental QALYs and ICER were all compared to the previous relevant strategy (i.e. not dominated). Note that lifetime costs, LYs, and QALYs are presented on average per person, while the number of infections (and reinfections after SVR) are cumulative for the whole cohort throughout the simulation between 2021 and 2065. SD, strongly dominated (more expensive and less or equally effective). SD, strongly dominated; ED, extendedly dominated; ICER, incremental cost-effectiveness ratio; QALY, quality-adjusted life year; SVR, sustained virological response. Strongly dominated = more expensive and less (or equally) effective. Extendedly dominated = at least one more expensive strategy has a lower ICER.

**Table S9. Sensitivity analysis assuming assortative mixing**

| Strategy | Lifetime costs  (€) | Life expectancy (LYs) | Quality-adjusted life expectancy (QALYs) | Number of infections  (reinfections after SVR) | ICER (€/LY) | ICER (€/QALY) |
| --- | --- | --- | --- | --- | --- | --- |
| S5 = screening every year; risk reduction (high-risk) | 351,315 | 20.2407 | 18.9945 | 2,068 (295) | - | - |
| S1 = screening every year (current practices) | 351,498 | 20.2405 | 18.9914 | 2,892 (450) | SD | SD |
| S6 = screening every year; risk reduction (all) | 351,520 | 20.2407 | 18.9946 | 2,049 (294) | SD | ED |
| S7 = screening every 6 months; risk reduction (high-risk) | 351,576 | 20.2407 | 18.9972 | 1,823 (257) | SD | 96,667 |
| S2 = screening every 6 months (current guidelines) | 351,707 | 20.2406 | 18.9954 | 2,444 (371) | SD | SD |
| S8 = screening every 6 months; risk reduction (all) | 351,781 | 20.2407 | 18.9973 | 1,808 (257) | SD | ED |
| S9 = screening every 3 months (high-risk); screening every 6 months (low-risk); risk reduction (high-risk) | 351,790 | 20.2407 | 18.9978 | 1,760 (248) | SD | 356,667 |
| S3 = screening every 3 months (high-risk); screening every 6 months (low-risk) | 351,912 | 20.2406 | 18.9962 | 2,333 (351) | SD | SD |
| S4 = screening every 3 months | 352,311 | 20.2406 | 18.9963 | 2,329 (351) | SD | SD |
| S10 = screening every 3 months; risk reduction (all) | 352,391 | 20.2407 | 18.9979 | 1,743 (247) | SD | 6,010,000 |

**Notes**: The model with assortative mixing was refitted (HCV transmission rate calibrated to reflect HCV incidence rates observed over 2014-2017). The strategies were sorted by increasing costs. Incremental costs, incremental QALYs and ICER were all compared to the previous relevant strategy (i.e. not dominated). Note that lifetime costs, LYs, and QALYs are presented on average per person, while the number of infections (and reinfections after SVR) are cumulative for the whole cohort throughout the simulation between 2021 and 2065. SD, strongly dominated (more expensive and less or equally effective). SD, strongly dominated; ED, extendedly dominated; ICER, incremental cost-effectiveness ratio; QALY, quality-adjusted life year; SVR, sustained virological response. Strongly dominated = more expensive and less (or equally) effective. Extendedly dominated = at least one more expensive strategy has a lower ICER.

# **S7. References**

1. R Core Team. R: A language and environment for statistical computing. R Foundation for Statistical Computing, Vienna, Austria. URL https://www.R-project.org/. 2018;

2. Castry M, Cousien A, Supervie V, Velter A, Ghosn J, Paltiel AD, et al. Impact of test-and-treat and risk reduction strategies on HCV transmission among MSM living with HIV in France: a modelling approach. Gut. 2021 Aug 1;70(8):1561.

3. Marin JM, Pudlo P, Robert CP, Ryder RJ. Approximate Bayesian computational methods. Stat Comput. 2012 Nov 1;22(6):1167–80.

4. Csilléry K, François O, Blum M. ABC: an R package for Approximate Bayesian computation (ABC). Methods in Ecology and Evolution. 2011 Jun 14;3.

5. Castry M, Cousien A, Bellet J, Champenois K, Pialoux G, Yazdanpanah Y, et al. Hepatitis C virus (HCV) incidence among men who have sex with men (MSM) living with HIV: results from the French Hospital Database on HIV (ANRS CO4-FHDH) cohort study, 2014 to 2017. Euro Surveill. 2021 Sep;26(38).

6. Berg R. The effectiveness of behavioural and psychosocial HIV/STI prevention interventions for MSM in Europe: A systematic review. Euro Surveill. 2009 Dec 3;14(48).

7. Henderson JT, Senger CA, Henninger M, Bean SI, Redmond N, O’Connor EA. Behavioral Counseling Interventions to Prevent Sexually Transmitted Infections: Updated Evidence Report and Systematic Review for the US Preventive Services Task Force. JAMA. 2020 Aug 18;324(7):682–99.

8. Johnson WD, Diaz RM, Flanders WD, Goodman M, Hill AN, Holtgrave D, et al. Behavioral interventions to reduce risk for sexual transmission of HIV among men who have sex with men. Cochrane Database Syst Rev. 2008 Jul 16;(3):CD001230.

9. Schwarzinger M, Deuffic-Burban S, Mallet V, Pol S, Pageaux GP, Canva-Delcambre V, et al. Lifetime costs attributable to chronic hepatitis C from the French healthcare perspective (ANRS N°12188). Journal of Hepatology. 2013 Apr;58:S21–2.

10. AFEF. Recommandations AFEF pour l’élimination de l’infection par le virus de l’hépatite C en France. 2018; Available from: https://afef.asso.fr/wp-content/uploads/2018/06/VF-INTERACTIF-RECO-VHC-AFEF-v2103.pdf

11. Papot E, Landman R, Louni F, Charpentier C, Peytavin G, Certain A, et al. Budget impact of antiretroviral therapy in a French clinic cohort. AIDS. 2017 Jun 1;31(9):1271–9.

12. Sloan CE, Champenois K, Choisy P, Losina E, Walensky RP, Schackman BR, et al. Newer drugs and earlier treatment: impact on lifetime cost of care for HIV-infected adults. AIDS. 2012 Jan 2;26(1):45–56.

13. Saeed YA, Phoon A, Bielecki JM, Mitsakakis N, Bremner KE, Abrahamyan L, et al. A Systematic Review and Meta-Analysis of Health Utilities in Patients With Chronic Hepatitis C. Value Health. 2020 Jan;23(1):127–37.

14. Pol S, Chevalier J, Branchoux S, Perry R, Milligan G, Gaudin AF. P0747 : Health related quality of life and utility values in chronic hepatitis C patients: A cross-sectional study in France, the Uk and Germany. Journal of Hepatology. 2015 Apr;62:S606.

15. Supervie V, Marty L, Lacombe JM, Dray-Spira R, Costagliola D, FHDH-ANRS CO4 study group. Looking Beyond the Cascade of HIV Care to End the AIDS Epidemic: Estimation of the Time Interval From HIV Infection to Viral Suppression. J Acquir Immune Defic Syndr. 2016 Nov 1;73(3):348–55.

16. Amele S, Peters L, Sluzhynska M, Yakovlev A, Scherrer A, Domingo P, et al. Establishing a hepatitis C continuum of care among HIV/hepatitis C virus-coinfected individuals in EuroSIDA. HIV Med. 2019 Apr;20(4):264–73.

17. Santé publique France. Bulletin de santé publique VIH/sida. DÉCOUVERTES DE SÉROPOSITIVITÉ VIH ET DIAGNOSTICS DE SIDA - FRANCE, 2018. 2019 [cited 2020 May 7]; Available from: https://www.santepubliquefrance.fr/maladies-et-traumatismes/infections-sexuellement-transmissibles/vih-sida/documents/bulletin-national/bulletin-de-sante-publique-vih-sida.-octobre-2019

18. Human Mortality Database. Human Mortality Database Web site. http://www.mortality.org. 2014. Accessed September 2019. 2018;

19. Bhaskaran K, Hamouda O, Sannes M, Boufassa F, Johnson AM, Lambert PC, et al. Changes in the risk of death after HIV seroconversion compared with mortality in the general population. JAMA. 2008 Jul 2;300(1):51–9.

20. Marty L, Cazein F, Panjo H, Pillonel J, Costagliola D, Supervie V, et al. Revealing geographical and population heterogeneity in HIV incidence, undiagnosed HIV prevalence and time to diagnosis to improve prevention and care: estimates for France. J Int AIDS Soc. 2018;21(3):e25100.

21. Pol S, Fouad F, Lemaitre M, Rodriguez I, Lada O, Rabiega P, et al. Impact of extending direct antiviral agents (DAA) availability in France: an observational cohort study (2015-2019) of data from French administrative healthcare databases (SNDS). Lancet Reg Health Eur. 2022 Feb;13:100281.

22. European Association for the Study of the Liver. EASL Recommendations on Treatment of Hepatitis C 2018. J Hepatol. 2018;69(2):461–511.

23. Piroth L, Wittkop L, Lacombe K, Rosenthal E, Gilbert C, Miailhes P, et al. Efficacy and safety of direct-acting antiviral regimens in HIV/HCV-co-infected patients - French ANRS CO13 HEPAVIH cohort. J Hepatol. 2017;67(1):23–31.

24. Piroth L, Larsen C, Binquet C, Alric L, Auperin I, Chaix ML, et al. Treatment of acute hepatitis C in human immunodeficiency virus-infected patients: the HEPAIG study. Hepatology. 2010 Dec;52(6):1915–21.

25. Thein HH, Yi Q, Dore GJ, Krahn MD. Natural history of hepatitis C virus infection in HIV-infected individuals and the impact of HIV in the era of highly active antiretroviral therapy: a meta-analysis. AIDS. 2008 Oct 1;22(15):1979–91.

26. Salmon-Ceron D, Nahon P, Layese R, Bourcier V, Sogni P, Bani-Sadr F, et al. Human Immunodeficiency Virus/Hepatitis C Virus (HCV) Co-infected Patients With Cirrhosis Are No Longer at Higher Risk for Hepatocellular Carcinoma or End-Stage Liver Disease as Compared to HCV Mono-infected Patients. Hepatology. 2019 Sep;70(3):939–54.

27. Deuffic-Burban S, Mathurin P, Pol S, Larsen C, Roudot-Thoraval F, Desenclos JC, et al. Impact of hepatitis C triple therapy availability upon the number of patients to be treated and associated costs in France: a model-based analysis. Gut. 2012 Feb;61(2):290–6.

28. Mourad A, Deuffic-Burban S, Ganne-Carrié N, Renaut-Vantroys T, Rosa I, Bouvier AM, et al. Hepatocellular carcinoma screening in patients with compensated hepatitis C virus (HCV)-related cirrhosis aware of their HCV status improves survival: a modeling approach. Hepatology. 2014 Apr;59(4):1471–81.

29. Salomon JA, Weinstein MC, Hammitt JK, Goldie SJ. Empirically calibrated model of hepatitis C virus infection in the United States. Am J Epidemiol. 2002 Oct 15;156(8):761–73.

30. Salomon JA, Weinstein MC, Hammitt JK, Goldie SJ. Cost-effectiveness of treatment for chronic hepatitis C infection in an evolving patient population. JAMA. 2003 Jul 9;290(2):228–37.

31. Agence de la Biomédecine. Rapport annuel 2019. Saint-Denis La Plaine. 2019;

32. Carrat F, Fontaine H, Dorival C, Simony M, Diallo A, Hezode C, et al. Clinical outcomes in patients with chronic hepatitis C after direct-acting antiviral treatment: a prospective cohort study. Lancet. 2019 Apr 6;393(10179):1453–64.

33. Tengs TO, Lin TH. A meta-analysis of utility estimates for HIV/AIDS. Med Decis Making. 2002 Dec;22(6):475–81.

34. Cossais S, Schwarzinger M, Pol S, Fontaine H, Larrey D, Pageaux GP, et al. Quality of life in patients with chronic hepatitis C infection: Severe comorbidities and disease perception matter more than liver-disease stage. PLoS One. 2019;14(5):e0215596.
